# Supplementary material for: Bioenergetic control of soil carbon dynamics across depth
Source: Nat Commun. 2022 Dec 12;13:7676. doi: 10.1038/s41467-022-34951-w (PMC9744916; doi:10.1038/s41467-022-34951-w)
Supplement: Supplementary file 1 — Supplementary Information [file 41467_2022_34951_MOESM1_ESM.pdf]

Supplementary Information

Bioenergetic control of soil carbon dynamics across depth

Ludovic Henneron[1,2*], Jerôme Balesdent[3,†], Gaël Alvarez[1], Pierre Barré[4], François Baudin[5], Isabelle Basile-Doelsch[3], Lauric Cécillon[2,4], Alejandro Fernandez-Martinez[6], Christine Hatté[7,8] and Sébastien Fontaine[1]

[1]UMR Ecosystème Prairial, INRAE, VetAgro Sup, Université Clermont Auvergne, Clermont-Ferrand, France
[2]ECODIV, Normandie Université, UNIROUEN, INRAE, Rouen, France
[3]CEREGE, Aix-Marseille Université, CNRS, INRAE, IRD, Aix en Provence, France
[4]Laboratoire de Géologie, Ecole normale supérieure, CNRS, Université PSL, IPSL, Paris, France
[5]ISTeP, Sorbonne Université, CNRS, Paris, France
[6]ISTerre, Université Grenoble Alpes, Université Savoie Mont Blanc, CNRS, IRD, IFSTTAR, Grenoble, France
[7]Laboratoire des Sciences du Climat et de l'Environnement, CEA, CNRS, UVSQ, Université Paris-Saclay, Gif-sur-Yvette, France
[8]Institute of Physics, CSE, Silesian University of Technology, Gliwice, Poland

Table of content

Supplementary methods
Soil biogeochemical properties 2-4
Radiocarbon (^{14}C) measurements and SOC turnover time estimation 2
Thermal analyses 2-4
Isotopic partitioning 4-7
Correction of plant-soil system respiration for background atmospheric CO_2 4
Plant-derived and soil-derived CO_2 fluxes 4-6
Living root biomass 6
Net rhizodeposition 7
Statistical analyses 7-8
Supplementary Figure 1. Energetic properties of soil organic matter across treatments 9
Supplementary Figure 2. Soil profiles and core sampling design across the three soil types 10
Supplementary Figure 3. Reduncancy analysis on soil biogeochemical drivers of SOC dynamics... 11
Supplementary Figure 4. Heatmap of partial correlations between variation in SOC dynamics parameters and soil organic matter properties across depth 12
Supplementary Figure 5. Soil core sampling and microcosm preparation method 13
Supplementary Figure 6. Relationship between turnover time and $\Delta^{14}C$ in year 2016 14
Supplementary Figure 7. X-ray diffractograms of samples from each soil horizon 15
Supplementary Table 1. Soil properties among soil types and layers 16
Supplementary Table 2. Statistical results for soil properties and incubations. 17
Supplementary Table 3. Statistical results for the first series of incubations 18
Supplementary Table 4. Statistical results for the second series of incubations 19
Supplementary Table 5. Statistical results for radiocarbon data in the second series of incubations. 20
Supplementary Table 6. Information about root C amount relative to SOC 20
Supplementary Table 7. Site characteristics for each soil type. 21
Supplementary Table 8. Additional soil properties. 21
Supplementary Table 9. Information about soil N fertilization for planted treatments 21
Supplementary Table 10. Plant biomass and $\delta^{13}C$ at the end of the experiment 22
Supplementary Table 11. Uncertainty in ^{13}C isotopic partitioning for the first incubation series 23
Supplementary Table 12. Uncertainty in ^{13}C isotopic partitioning for the second incubation series . 24
Supplementary Table 13. Uncertainty in ^{14}C isotopic partitioning for the second incubation series . 25
Supplementary Table 14. Uncertainty in ^{13}C isotopic partitioning of SOC 25
Supplementary References 26-28

Supplementary methods

Soil biogeochemical properties

Radiocarbon (^{14}C) measurements and SOC turnover time estimation

Once in the ^{14}C laboratory, soil samples were crushed under 200 µm to homogeneize it. As no carbonate was expected, samples were not acidified prior to the measurement. According to SOC content of the samples, ^{14}C measurements were performed using either a solid source for topsoil samples or gas source for subsoil samples. Aliquotes were sampled in tin capsules to get either 1 mg of SOC for topsoils or twice 70 µg of SOC for subsoils. For topsoil samples, CO_2 evolved from SOC was graphitized using an automated AGE 3 graphitization device[1], and ^{14}C measurement was performed on *ECHo*MICADAS[2–4] through the solid source. For each subsoil samples, two ^{14}C measurements were performed on *ECHo*MICADAS through the gaz source connected by a Gas Ion Source (GIS)[5] to an elementar analyser (EA) evolving SOC to CO_2. The ^{14}C signature of root biomass were performed as decribed above for topsoil samples. Barium carbonate from soil respired CO_2 was transformed into CO_2 in a semi-automated carbonate line[6]. Evolved CO_2 was sealed under vacuum in pyrex microtube and introduced into the ECHoMICADAS gas source through the cracking-GIS interface[5]. All ^{14}C results have been corrected for mass-dependent isotopic fractionation using AMS-derived ^{13}C measurements, and were expressed as deviations from the absolute (decay-corrected) Oxalic Acid I (OX1) standard (Δ^{14}C, in ‰)[7,8]. The average measurement precision of the Δ^{14}C values was 2.4 ‰.

We estimated SOC turnover time based on radiocarbon measurements using a modelling approach. The following time-dependent, homogeneous one-pool model[7,9] was used:

$$\left(F_{SOC}^{14c} \times SOC\right)_t = \left(I \times F_{atm}^{14c}\right)_{t-Tr} + \left(F_{SOC}^{14c} \times SOC\right)_{t-1}\left(1 - \frac{1}{\tau} - \lambda\right) \quad (1)$$

$$\text{given } F^{14c} = \left(\frac{\Delta 14c}{1000}\right) + 1$$

where at time *t*, $F^{14C}{}_{SOC}$ is the ^{14}C content of SOC, SOC is the SOC stock, I is the rate of C input from the atmosphere to SOC, $F^{14C}{}_{atm}$ is the ^{14}C content of CO_2 in the local atmosphere, T_R is the mean C transit time through living plant material, τ is the mean SOC turnover time and λ is the radioactive decay constant for ^{14}C (1.21×10^{-4} year^{-1}). Assuming T_R = 1 year and SOC stocks to be at steady-state so that $C_t = C_{t-1} = I \times \tau$, the equation (1) reduces to:

$$F_{SOC,t}^{14c} = \frac{1}{\tau} F_{atm,t-1}^{14c} + F_{SOC,t-1}^{14c}\left(1 - \frac{1}{\tau} - \lambda\right) \quad (2)$$

where *t*-1 is the year preceding time *t*. We ran the model from 50 kyr BP until the year 2016 using the 'SoilR' package to calculate the predicted SOC Δ^{14}C at the year of sampling for a range of τ values (1 to 30,000 years)[10]. We used Δ^{14}C atmospheric values from Reimer et al., (2020)[11] for the period 0–50 000 years BP, from Hua et al., (2013)[12] for the period 1950–2010, and we calculated extrapolated values using an exponential smoothing state–space modeling approach for the period 2010–2016[10]. We then derived τ values from our Δ^{14}C measurements for each sample based on the relationship between τ and predicted Δ^{14}C (Supplementary Fig. 6).

Thermal analyses

Rock-Eval® thermal analysis consisting in evolved gas analysis during ramped combustion was performed using a Rock-Eval® 6 Turbo device (Vinci Technologies, France) following a procedure adapted for SOM analysis[13] to measure the activation energy (E_a) of thermal SOC decomposition. Briefly, ca. 60 mg of ground (<250 µm) samples were subjected to sequential pyrolysis and oxidation phases. The pyrolysis phase was carried out in an N_2 atmosphere with a 3 min isotherm at 200 °C followed by a temperature ramp from 200 to 650 °C at a heating rate of 30 °C min^{-1}. The oxidation phase was carried out in laboratory air atmosphere with a 1 min isotherm at 300 °C followed by a temperature ramp from 300 to 850 °C at a heating rate of 20 °C min^{-1} and a final 5 min isotherm at 850 °C. Hydrocarbon effluents (H_xC_y) were quantified

by flame ionization detection during the pyrolysis phase, while CO and CO_2 were quantified by infrared detection during both ramping phases. Each Rock-Eval® thermal analysis generated five thermograms corresponding to hydrocarbon effluents (H_xC_y-pyrolysis thermogram), CO (CO-pyrolysis thermogram) and CO_2 (CO_2-pyrolysis thermogram) measured at each second during the pyrolysis phase, and to the CO (CO-oxidation thermogram) and CO_2 (CO_2-oxidation thermogram) measured at each second during the oxidation phase. Thermograms were integrated on different time intervals depending on the thermogram. The integration omitted the first 200 seconds of the analysis for the three thermograms of the pyrolysis phase. The integration ended at the time of analysis corresponding to the maximum oven temperatures of 650 °C (H_xC_y-pyrolysis thermogram), 560 °C (CO-pyrolysis and CO_2-pyrolysis thermograms), 850 °C (CO-oxidation thermogram) and 611 °C (CO_2-oxidation thermogram). These intervals of integration prevented any interference by inorganic carbon from most soil carbonates[14]. Before determination of the activation energy (E_a) of SOC decomposition, the three thermograms of the pyrolysis phase and the two thermograms of the oxidation phase were combined into single thermograms of mass-equivalent C evolved during each ramping phase[15].

Assuming first-order reaction kinetics during ramped combustion, we used a regularized, inverse method to determine the continuous distribution of E_a that best predicts the measured SOC decay profile[16]. Thermograms of mass-equivalent C evolved during both pyrolysis and oxidation were analysed separately using the 'rampedpyrox' Python package[17], resulting in distinct E_a distributions for each ramping phase. For both the thermograms and E_a distributions, the data from each phase were then merged by averaging weighted based on the relative amount of C evolved during each ramping phase. Each combined continuous E_a distribution was then integrated to calculate the mean (μE_a) and standard deviation (σE_a) of activation energy (kJ mol^{-1} SOM)[16]. The E_a represents the energy input required for SOC combustion and was used here as a proxy for the energetic barriers to microbial SOC decomposition, that is the energy input needed from microbes producing exoenzymes to access and metabolize it[18]. We acknowledge that these computed E_a values of thermal SOC decomposition are expected to be much higher than for naturally occurring SOC biodegradation catalysed by microbial exoenzymes[19]. However, many studies combining radiocarbon and thermal analyses have found E_a of SOC combustion to be strongly related to SOC biogeochemical stability, with increasing E_a for SOC having increasing SOC radiocarbon ages[20–22]. We thus assumed here that the E_a of SOC combustion remains a good proxy of the energy investment in exoenzymes production needed for microbial decomposers to acquire this SOC. Though the pyrolysis phase may have overestimated our E_a values due to charring effects[23], it has been found that these charring reactions do not affect the determination and interpretations of relative thermal stability of SOC[24]. Previous studies further showed that ramped pyrolysis and oxidation yielded similar E_a results[20,21].

Two standard Rock-Eval® 6 parameters describing SOM bulk chemistry in term of hydrogen and oxygen composition were determined[14], that are the hydrogen and oxygen indices (HI and OI). The HI index was calculated the amount of hydrocarbons (H_xC_y) formed during thermal pyrolysis of the sample between 200 and 650 °C divided by the total SOC of the sample. The OI was calculated using the following equation[25]:

$$\mathrm{OI} = \frac{16}{28} \times \mathrm{OI_{CO}} + \frac{32}{44} \times \mathrm{OI_{CO2}} \quad (3)$$

where OI_{CO2} and OI_{CO} correspond respectively to the CO_2 and CO yielded during thermal pyrolysis of the sample between respectively 200 and 400 °C (OI_{CO2}) and 200 and 550 °C (OI_{CO}) divided by the total SOC of the sample. The HI and OI indices are well correlated respectively with elemental H:C and O:C ratios[26]. The analysis of biologically relevant standards showed, for example, that lipids have high HI values whereas polyphenols and carbohydrates have higher OI values[27]. A preliminary study measuring the elemental H:C and O:C ratios of SOM based on nuclear magnetic resonance spectroscopy coupled to a molecular

mixing model found strong relations with respectively HI (H:C = 1.21 + HI × 8.20 × 10^{-5}, r^2 = 0.53) and OI (O:C = 0.35 + OI × 1.27 × 10^{-3}, r^2 = 0.46). We used these equations to estimate the elemental H:C and O:C ratios of SOM, which allowed us to then calculate the SOC molar mass (M_{SOC}, g SOC mol^{-1} SOM) using the following equation:

$$M_{SOC} = M_C + \frac{M_H}{H:C} + \frac{M_O}{O:C} + \frac{M_N}{N:C} \quad (4)$$

where M_C, M_H, M_O and M_N are respectively the molar mass (g mol^{-1}) of C, H, O and N.

Differential scanning calorimetry (DSC) during ramped combustion was also performed to measure the net energy released by SOM combustion (enthalpy of combustion), knowing that some of the energy applied to the sample is consumed by the breakdown of the organo-mineral associations. The mineral phase itself could also contribute to heat flux during ramped combustion, mainly generating endothermic reactions. However, previous studies showed that the contribution of the mineral phase to heat flux is usually small relatively to the contribution from SOM[28,29]. Briefly, ca. 50 mg of ground (<250 µm) samples were placed in 70 µL alumina crucibles, with an identical empty crucible used as a reference, and subjected to oxidation ramping (25–1000 °C, ramping rate of 5 °C·min^{-1} under a synthetic CO_2-free air atmosphere) using a thermal analyser simultaneously performing DSC and thermogravimetry analyses (TGA/DSC 3+ model, Mettler-Toledo, Greifensee, Switzerland). DSC heat fluxes (the exothermic or endothermic energy fluxes from the sample, referenced to an empty alumina crucible) were recorded every second, and the DSC thermograms were corrected *a posteriori* using a spline linear baseline. Net energy released was determined by integrating the exothermic region of the DSC thermogram (185-600 °C, Figure S3c), which represents the temperature range in which SOM is combusted[28]. Energy density of SOM (ΔE, in kJ g^{-1} SOC, Supplementary Table 8) was calculated as the net energy released divided by SOC content, which was recovered from thermogravimetry mass loss converted to SOC content with an equation accounting for mass loss due to clay water loss[30]. To get both ΔE and μE_a expressed in the same unit, ΔE was converted in kJ mol^{-1} SOM by multiplying it with the SOC molar mass (g SOC mol^{-1} SOM) estimated based on C:H:O:N stoichiometry as explained above. We acknowledge that our SOC molar mass values remain approximative because of the uncertainty associated to the estimation of H:C and O:C ratios based on HI and OI indices. However, this allowed ΔE and μE_a to be expressed in the same unit for the ROI calculation, and the estimated SOC molar mass varied little between treatments (Supplementary Table 8).

Isotopic partitioning

Correction of plant-soil system respiration for background atmospheric CO_2

During the sampling of CO_2 fluxes in the first series of incubations, the microcosms were sealed in opaque, airtight PVC chambers. Just before the sealing, each PVC chamber was intensively ventilated for 1 min with ambient air and we took care to avoid any contamination of the chamber air by breathing. The ambient air used for the ventilation was sampled to measure the initial amount and $\delta^{13}C$ of CO_2 in the chamber at the beginning of the incubation. Each microcosm sealed in chamber was then incubated for 24 h at temperature-controlled conditions (21.5 °C) in the laboratory. After 24 h of CO_2 release by the plant-soil system, the chamber gas was sampled by transfer into a glass flask with a vacuum pump. After beeing ventilated ten times his volume the flask was airtight sealed until gas analysis. Its CO_2 concentration as well as $\delta^{13}C$ were measured using a Gas Chromatograph (Clarus 480, Perkin Elmer, Waltham, MA, USA) and an isotope laser spectrometer (G2201-i Isotopic Analyser, Picarro, Santa Clara, CA, USA). The amount and $\delta^{13}C$ of CO_2 derived from the plant-soil system respiration were corrected for background atmospheric CO_2 using the following equations:

$$R_{total} = {CO_2}_{chamber} - {CO_2}_{atmosphere} \quad (5)$$

$$\delta^{13}C_{total} = \frac{({CO_2}_{chamber} \times \delta^{13}C_{chamber}) - ({CO_2}_{atmosphere} \times \delta^{13}C_{atmosphere})}{R_{total}} \quad (6)$$

where R_{total} and $\delta^{13}C_{total}$ are the total amount and $\delta^{13}C$ of CO_2 release by the plant-soil system after correction; $CO_{2\text{-}chamber}$ and $\delta^{13}C_{chamber}$ are the total amount and $\delta^{13}C$ of CO_2 measured in the chamber at the end of the incubation; and $CO_{2\text{-}atmosphere}$ and $\delta^{13}C_{atmosphere}$ are the total amount and $\delta^{13}C$ of atmospheric CO_2 sampled at the beginning of the incubation.

Plant-derived and soil-derived CO_2 fluxes

In the first series of incubations, we observed that the CO_2 derived from the respiration of the unplanted microcosms was systematically depleted in ^{13}C relative to the $\delta^{13}C$ of SOC for most treatments (Supplementary Table 11), with an average differences in $\delta^{13}C$ of -4.2 ‰, whereas CO_2 derived from SOC respiration usually tend to be slightly ^{13}C enriched relative to SOC[31]. We concomitantly observed algae development on the soil surface of unplanted microcosms. Furthermore, Cros, et al. (2019)[32] observed the fixation of a small quantity of CO_2 in unplanted soil linked to the development of algae during daytime. They also observed a depleted $\delta^{13}C$ signature of unplanted soil respiration during 24 h dark incubations, which was of the same order of magnitude relative to their $\delta^{13}C$ of SOC (~ -3 to -4 ‰). This highlights that a non-negligible photosynthetic activity occurred in the unplanted soil due to algae development, which could in turn respire labeled OC and explain the depleted $\delta^{13}C$ of the unplanted soil respiration. Following the procedure used in a previous study[33], we thus decided to apply the same isotopic partitioning on R_{total} of unplanted microcosms to correct for algae respiration, thus yielding more accurate estimation of R_{soil} for the unplanted controls. The $\delta^{13}C$ values of unplanted soil respiration measured at the end of experiment in the second series of incubations after removing the thin layer contaminated by algae on soil surface (~ 1 to 2 mm) were thus used as $\delta^{13}C_{soil}$ for both planted and unplanted soil in the first series of incubations.

Cros et al. (personal communication) also tested the possibility this depleted $\delta^{13}C$ could be due to labelled CO_2 back-diffusion during the 24 h dark incubation. After 24 h of pot ventilation either with labeled or with unlabeled (ambient) air, they measured the $\delta^{13}C$ of the CO_2 released during the incubation. In both cases, they found very similar $\delta^{13}C$, indicating that the contribution of labelled CO_2 back-diffusion to the depleted $\delta^{13}C$ signature of unplanted soil respiration was negligible. This is consistent with a recent study that also found very weak back-diffusion of labelled CO_2 during incubation[34].

Uncertainty in ^{13}C source partitioning related to sampling and analytical errors was calculated following Phillips & Gregg (2001)[35] with the following equation:

$$\sigma_{f_{soil}} = \sqrt{\frac{1}{(\bar{\delta}_{soil} - \bar{\delta}_{plant})^2}\left[\sigma^2_{\bar{\delta}_{total}} + f^2_{soil}\sigma^2_{\bar{\delta}_{soil}} + (1 - f_{soil})^2\sigma^2_{\bar{\delta}_{plant}}\right]} \quad (10)$$

given $f_{soil} = \frac{\bar{\delta}_{total} - \bar{\delta}_{plant}}{\bar{\delta}_{soil} - \bar{\delta}_{plant}}$ and $\sigma^2_{\bar{\delta}_x} = \frac{\sigma^2_{\delta_x}}{n_x}\frac{\sigma^2_{analytical}}{n_x}$

where σ_{fsoil} is the standard error of the mean soil source proportion, that is f_{soil}; $\bar{\delta}_{total}$, $\bar{\delta}_{soil}$, $\bar{\delta}_{plant}$ are respectively the mean $\delta^{13}C$ of the mixture and of soil and plant sources; $\sigma_{\bar{\delta}_{total}}$, $\sigma_{\bar{\delta}_{soil}}$ and $\sigma_{\bar{\delta}_{plant}}$ are respectively the standard error of mean $\delta^{13}C$ of the mixture and of soil and plant sources; σ_{δ_x} is the population standard deviation in $\delta^{13}C$ among individual samples of source *x; $\sigma_{analytical}$* is the $\delta^{13}C$ analytical standard deviation; and n_x is the population size of source *x*. The $\sigma_{analytical}$ values are respectively 0.10 and 0.12 ‰ for elemental analyser/isotope-ratio mass spectrometer and isotope laser spectrometer measurements. Since isotopic partitioning was performed at the microcosm level where the whole plant biomass has been sampled (plant source) and the isotopic signature has been measured on a well homogenized sample for both microcosm atmosphere (mixture) and plant biomass (plant source), the σ_{δ_x} values for these two sources were assumed to be zero. We found that σ_{fsoil} *values* were on average 1.1 and 1.2 % respectively for the first and second series of incubations (Supplementary Tables 11 and 12), indicating low level of uncertainty associated with sampling and analytical errors[35].

Our isotopic partitioning relied on several assumptions about the isotopic signature of our sources. For the first series of incubations, we used the mass-weighted $\delta^{13}C$ of the mesocosm shoot and living root biomass as $\delta^{13}C_{plant}$, assuming negligible fractionation during whole-plant respiration[36]. We also used the mean $\delta^{13}C$ of plant biomass across all treatments as $\delta^{13}C_{plant}$ for unplanted soil, assuming similar $\delta^{13}C$ fractionation during C3 photosynthesis for algae than for *D. glomerata*[37]. Finally, we used the $\delta^{13}C$ values of unplanted control respiration measured at the end of experiment in the second series of incubations as $\delta^{13}C_{soil}$ for both planted and unplanted soil in the first series of incubations, assuming constant $\delta^{13}C$ fractionation of soil-derived respiration across time. For the second series of incubations, we used $\delta^{13}C_{plant}$ values taken as the living root biomass $\delta^{13}C$ values corrected by the $\delta^{13}C$ fractionation factor (f) of root respiration, assuming a constant f value of -0.61 ‰[33]. We also used the $\Delta^{14}C$ of root biomass as $\Delta^{14}C_{plant}$, assuming no fractionation of ^{14}C during respiration of root-derived OC.

In order to evaluate the uncertainty associated with our isotopic mixing model assumptions, we performed a sensitivity analysis where we quantified the error in f_{soil} and $\Delta^{14}C_{soil}$ related to a 1 ‰ variation in $\delta^{13}C_{soil}$ and $\delta^{13}C_{plant}$, and a 2 ‰ variation in $\Delta^{14}C_{plant}$. Given that the deviation can be positive or negative, we tested a deviation of +0.5 or +1 and −0.5 or −1 ‰ respectively corresponding to an amplitude of 1 or 2 ‰. The error was then expressed as the difference in f_{soil} and $\Delta^{14}C_{soil}$ between +0.5 or +1 and −0.5 or −1 ‰ source values. We found that the average errors in f_{soil} related to a 1 ‰ variation in $\delta^{13}C_{plant}$ was respectively 2.0 and 1.9 % for the first and second series of incubations, while the average error related to a 1 ‰ variation in $\delta^{13}C_{soil}$ was 1.5 % for the first series of incubations (Supplementary Tables 11 and 12). We also found that the average errors in the absolute values of RPE related to a 1 ‰ variation in $\delta^{13}C_{plant}$ was respectively 19.3 and 12.2 % for the first and second series of incubations, while the average error related to a 1 ‰ variation in $\delta^{13}C_{soil}$ was 0.2 % for the first series of incubations (Supplementary Tables 11 and 12). The average errors in $\Delta^{14}C_{soil}$ related to a 1 ‰ variation in $\delta^{13}C_{plant}$ and a 2 ‰ variation $\Delta^{14}C_{plant}$ were respectively 19.4 and 2.8 ‰ (Supplementary Table 13). These low levels of uncertainty provide evidence that our isotopic partitioning was robust.

For the first and second series of incubations, the average $\delta^{13}C$ difference between plant and soil sources were respectively 24.9 and 24.8 ‰ (range of respectively 23.6 to 26.4, and 22.9 to 26.6 across treatments). This is substantially larger than source difference typical yielded by the natural ^{13}C-labeling method[38], as well as the traditional continuous ^{13}C-labeling method based on adding fossil fuel-derived CO_2 in a flow of ambient air[39]. This strong labeling allows to improve the accuracy of isotopic partitioning[31,32,35].

Living root biomass

As the root material harvested for topsoil after the second incubation series was composed of both pre-existing root litter (unlabelled) and living root (labelled) that could not be clearly visually sorted, we used an isotopic partitioning method to estimate the biomass of living roots for each planted topsoil core. Assuming negligible pre-existing (unlabeled) root litter biomass in subsoil at the end of the experiment and equal $\delta^{13}C$ difference between shoot and root biomass ($\Delta\delta^{13}C_{shoot\text{-}root}$) in topsoil and subsoil, we calculated the $\delta^{13}C$ of living root biomass as the microcosm shoot $\delta^{13}C$ minus the $\Delta\delta^{13}C_{shoot\text{-}root}$ of subsoil (Supplementary Table 10). Further assuming equal $\delta^{13}C$ for pre-existing (dead) root litter and SOC, we used the following equation based on a two-source isotopic mixing model.

$$Root_{living} = Root_{total} \times \frac{\delta^{13}C_{total} - \delta^{13}C_{dead}}{\delta^{13}C_{living} - \delta^{13}C_{dead}} \quad (11)$$

where $Root_{total}$ and $\delta^{13}C_{total}$ are respectively the biomass and $\delta^{13}C$ of both dead and living roots; $Root_{living}$ and $\delta^{13}C_{living}$ are respectively the biomass and $\delta^{13}C$ of both living roots; and $\delta^{13}C_{dead}$ is the $\delta^{13}C$ of dead roots.

The standard errors in ^{13}C source partitioning related to sampling and analytical errors was on average 0.6 %, indicating low uncertainty level (Supplementary Table 10). A similar

sensitivity analysis performed as described above showed that the average errors in ^{13}C source partitioning related to a 1 ‰ variation in $\delta^{13}C_{living}$ was 3.7 %, while the average error related to a 1 ‰ variation in $\delta^{13}C_{dead}$ was 0.6 % (Supplementary Table 10). These low levels of uncertainty provide evidence that our isotopic partitioning was here also robust.

Net rhizodeposition

We quantified the net rhizodeposition corresponding to the root-derived SOC remaining in the soil after microbial utilization. Net rhizodeposition was used here as a proxy of the gross rhizodeposition corresponding to the flux of fresh OC supply into the soil via living roots, which remains so far very challenging to quantify[40]. We acknowledge that net rhizodeposition is not only driven by gross rhizodeposition, but also by other factors affecting the stabilization and destabilization of rhizodeposits such as soil mineralogy or microbial communities. However, we argue that net rhizodeposition remains a good proxy of gross rhizodeposition at the microcosm scale, and provides here useful insights about how SOC decomposition response to variation in living root density with core depth in the second incubation series was related to variation in gross rhizodeposition.

Net rhizodeposition was estimated for each planted soil core harvested after the second incubation series using the following equation based on a two-source isotopic mixing model:

$$Net\ rhizodeposition = SOC_{total} \times \frac{\delta^{13}C_{SOC-final} - \delta^{13}C_{SOC-initial}}{\delta^{13}C_{root} - \delta^{13}C_{SOC-initial}} \qquad (12)$$

where SOC_{total} and $\delta^{13}C_{SOC\text{-}final}$ are respectively the SOC content and $\delta^{13}C$ of the planted soil core at the end of the experiment, $\delta^{13}C_{SOC\text{-}initial}$ is the average $\delta^{13}C$ of SOC from soil cores sampled in the field at the beginning of the experiment, and $\delta^{13}C_{plant}$ is the $\delta^{13}C$ of living root biomass of the planted microcosm.

A sensitivity analysis was performed as described above to assess the uncertainty associated to our assumption of a negligible ^{13}C fractionation of root-derived OC during microbial utilization. The average errors in ^{13}C source partitioning related to a 1 ‰ variation in $\delta^{13}C_{root}$ was only 0.06 %, indicating low levels of uncertainty related to this assumption (Supplementary Table 14). However, the standard error in ^{13}C source partitioning related to sampling and analytical errors was on average 0.77 %, which we acknowledge is rather high relative the average proportion of root-derived SOC found at the end of the experiment (1.37 %, Supplementary Table 14). Such level of uncertainty nevertheless remains within the range of previous experiments quantifying plant-derived OC in a large reservoir of existing SOC with a comparable labeling intensity[33,41–43].

Statistical analyses

We used a rotated principal component analysis to explore soil properties covariance and divergence divergence between treatments (Fig. 1a). The rotated principal component analysis (RCA) was performed using the '*principal*' function of the '*Psych*' package[44]. Rotation is commonly used in principal component analysis to simplify interpretation of principal components by maximizing/minimizing the correlations between factors and component axes. In order to simplify the RCA ordination, we selected only two axes. Analyses of variance were used to partition the variance explained by the factors 'soil layer', 'soil type' and their interaction in the two first axis scores (Fig. 1b) and soil properties (Supplementary Table 2).

Partial η^2 of depth effect on SOC properties is calculated as the sum of squares for the depth effect divided by the total sum of squares (after accounting for the variance associated with soil type effect). It was computed using the '*eta_squared*' function of the '*effectsize*' package[45] on a linear mixed-effect model fitted using the '*lmer*' function of the '*lme4*' package[46] and including 'soil layer' as a fixed factor and 'soil type' as a random factor.

For each series of incubations, the responses of k_{SOC}, RPE and $\Delta^{14}C_{soil}$ to predictors were assessed by ordinary least squares regression for each treatment (Figs. 3, 4 and 5). We tested

linear (Y = a + bX), polynomial (Y = a + bX + cX^2) and power (Y = aX^b) regression functions, where Y is the response variable and X is the predictor. The k_{SOC} and RPE values were standardized for each treatment to a common high value of the following predictors: 'respiration of plant-derived OC' for the first incubation series, as well as 'living root density', 'respiration of plant-derived OC' and 'net rhizodeposition' for the second incubation series. To do so, we used the regression model parameters to predict k_{SOC} and RPE values at the mean predictor value across treatments of the last sampling time in the first incubation series, corresponding to 9.94 g C-CO_2 m^{-2} day^{-1} for 'respiration of plant-derived OC'. For the second incubation series, the same procedure was applied with the mean predictor values across treatments in the 0-20 cm depth soil core, corresponding to 3.57 g dm^{-3} for 'living root density', 20.48 mg C-CO_2 dm^{-3} day^{-1} for 'respiration of plant-derived OC' and 0.79 g SOC_{root} dm^{-3} for 'net rhizodeposition'. Additionally, we used analyses of covariance including the quantitative explanatory variables, the factors 'soil layers' and 'soil type', and their interactions as fixed factors to test their effects and quantify the proportion of variance they explain (Supplementary Tables 3, 4 and 5). To deal with the repeated measures design in both the first and second incubation series, we used linear mixed-effect models including 'microcosm' as random factor in regression and analyses of covariance. Linear mixed-effects models were fitted using the '*lmer*' function of the '*lme4*' package[46]. Statistical significance of fixed predictors were assessed based on Satterthwaite's approximation of denominator degrees of freedom using the '*anova*' function of the '*lmerTest*' package[47]. Marginal and conditional r^2 were computed based on Nakagawa and Schielzeth[48] using the '*r.squaredGLMM*' function of the '*MuMIn*' package[49].

To examine the relationship between soil biogeochemistry and organic matter dynamics, we explore partial bivariate relationships between variation in SOC dynamics and SOM properties across depth while controlling for soil types. Before quantification of bivariate relationships, each variable was centered using the residuals of a linear model with 'soil type' as a fixed effect. Partial bivariate relationships of radiocarbon-based mean SOC age with both the return-on-energy-investment of microbial SOC decomposition (ROI) and root density were first examined using ordinary least squares linear regression models (Fig. 2). The slope coefficients of the regression models were standardized by range. To do so, the unstandardized slope coefficient was multiped by the range (the difference between the maximum and minimum values) of the predictor and divided by the range of the dependant variable). It was computed using the '*coefs*' function of the '*piecewiseSEM*' package[50] Partial correlations between SOC dynamics variables, including ^{14}C-based SOC turnover time as well as unplanted k_{SOC} and standardized RPE of each incubation series, and SOM properties were then also performed by computing Spearman's correlation coefficients (Supplementary Fig. 3). It was computed using the '*rcorr*' function of the '*Hmisc*' package[51] with depth-centered variables. Additionally, we performed an ordination of the same SOC dynamics variables constrained by soil biogeochemical predictors (same set of variables used in the RCA) using a redundancy analysis (Supplementary Fig. 4). The redundancy analysis (RDA) was performed using the '*rda*' function of the '*vegan*' package[52] with raw variables. The significance of the overall constrained ordination and of the two first axes were tested by permutation tests[53], using the '*anova*' function of the '*vegan*' package[52] (1,000 permutations).

All analyses were performed using R v3.4.3[54]. Null hypothesis testing was always based on two-sided statistical tests. The normal distribution and homogeneity of variances of the model residuals were graphically checked and data were log-transformed when necessary.

Supplementary Figures

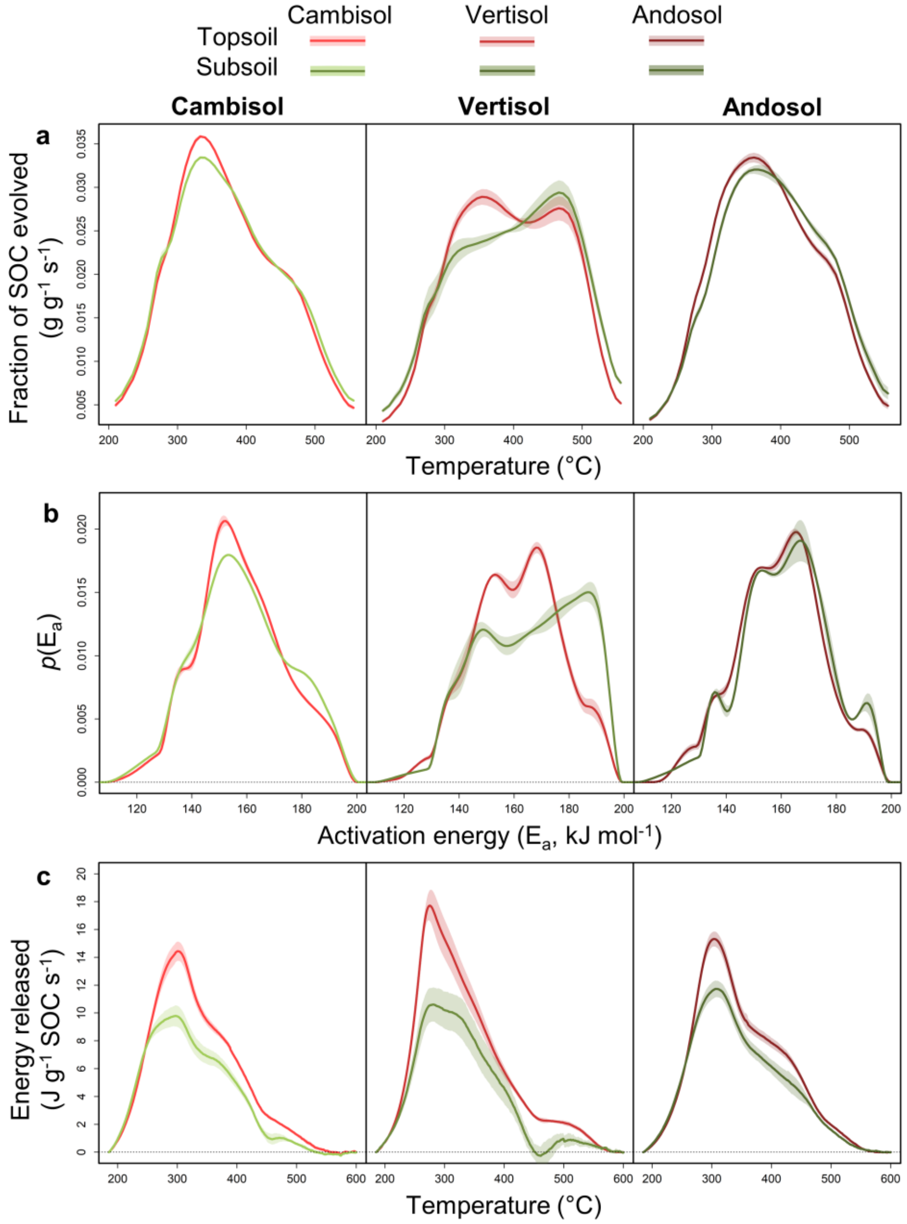

Supplementary Fig. 1. Energetic properties of soil organic matter across treatments. **a**, thermogram of soil organic carbon (SOC) decomposition measured by Rock-Eval sequential pyrolysis and oxidation. **b**, distribution of activation energy, $p(E_a)$. **c**, thermogram of heat flow measured by differential scanning calorimetry. Polygons around lines represent 95% confidence intervals around treatment means (n = 4 replicate soil cores).

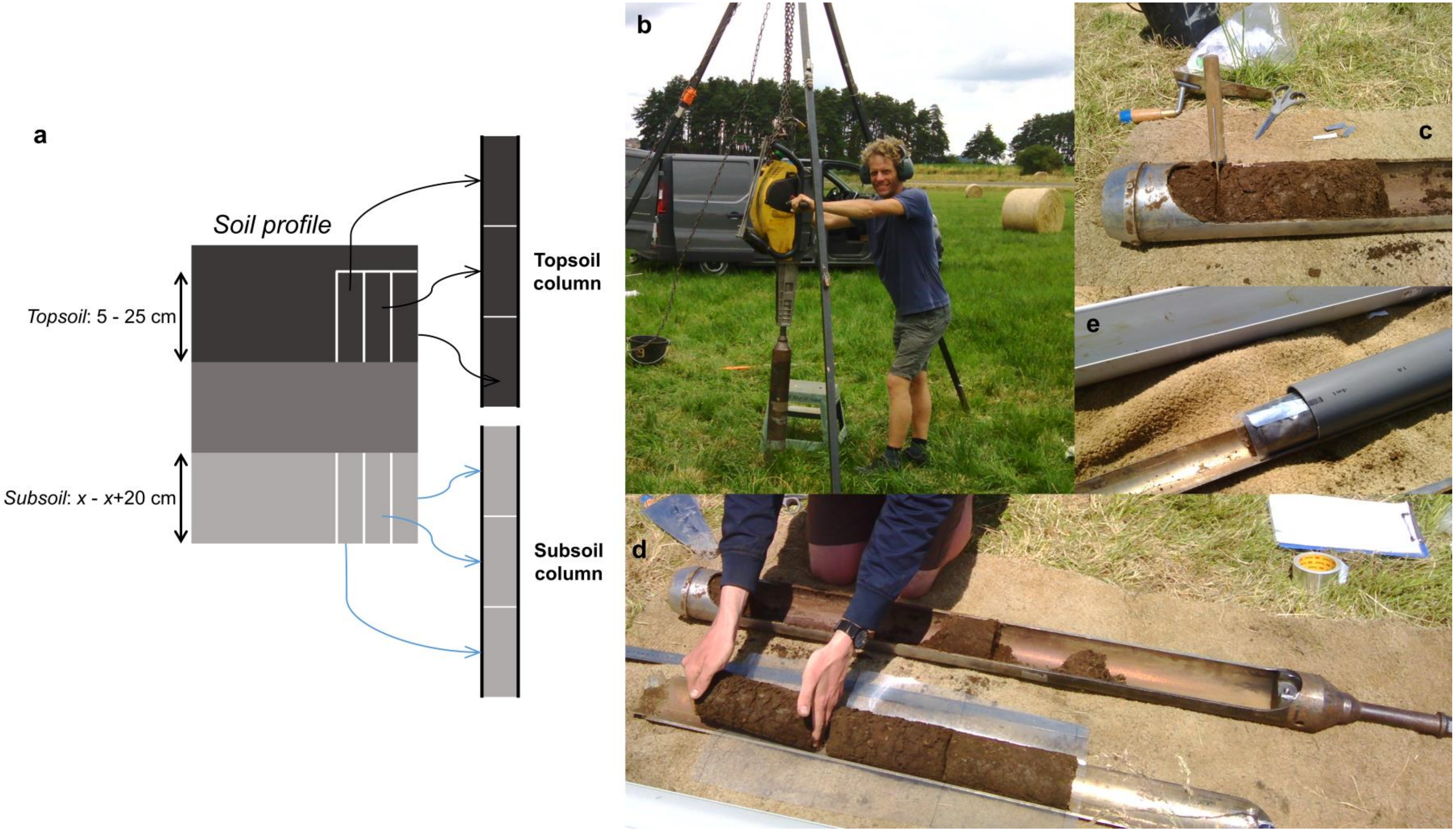

Supplementary Fig. 2. Soil core sampling and microcosm preparation method. A percussion core drill equipped with a steel tube that can be opened from sideways was used to extract intact soil columns of 8 cm diameter (**a**, **b**, **d**). For each layer, three soil cores collected with a knife (**c**) from the same depth in the initial soil column were gently stacked together (**a**, **d**), tightly sealed within a polyethylene sheath (**d**, **e**) and transferred into a bottom-capped polyvinyl chloride (PVC) tube (**e**) to form a new soil column exclusively made of topsoil or subsoil (**a**). See Supplementary Fig. 5 for the soil core sampling design of each soil type.

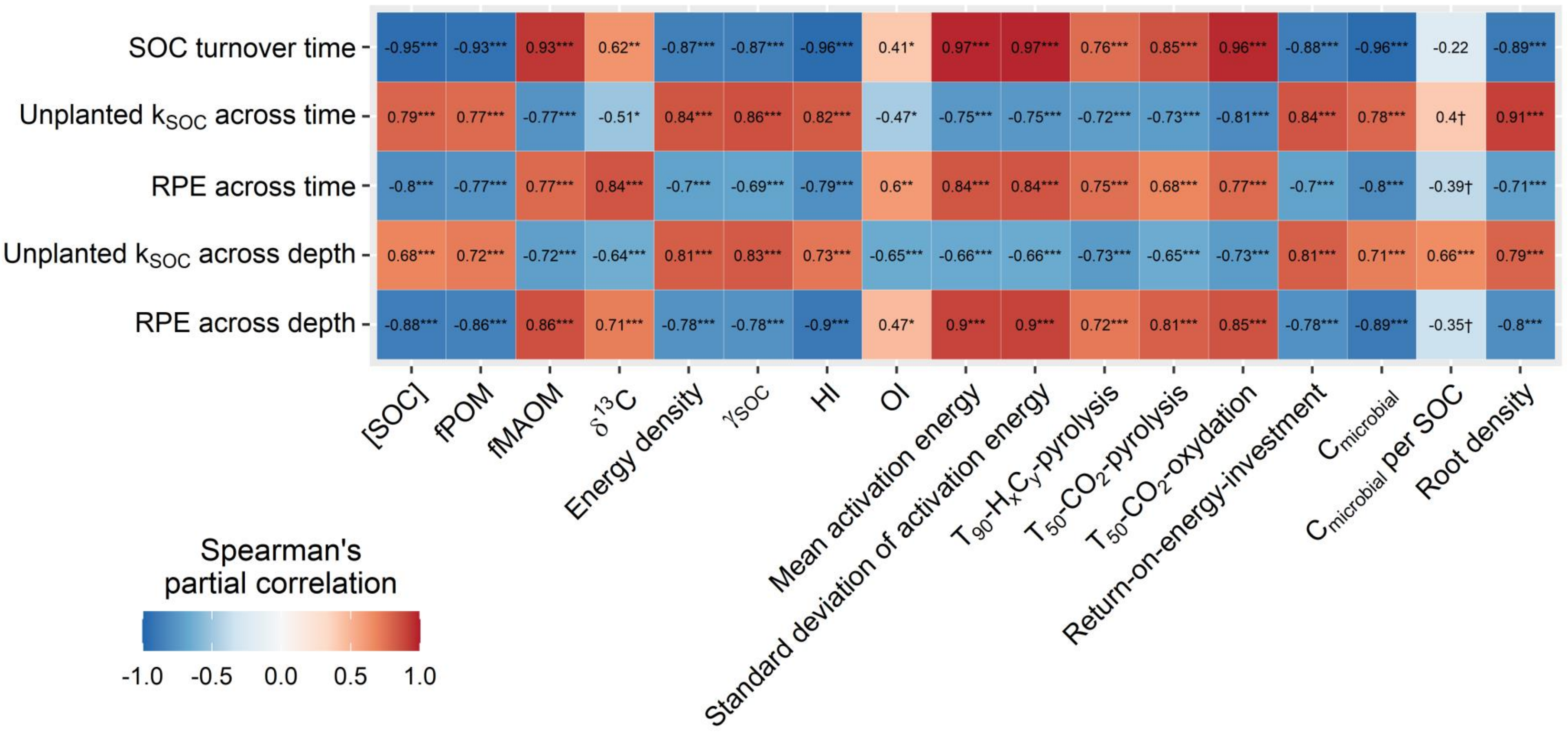

Supplementary Fig. 3. Heatmap of partial correlations between variation in soil organic carbon (SOC) dynamics parameters and soil organic matter (SOM) properties across depth (n = 24 soil cores/microcosms). Each variable was normalized for variation across soil type using the residuals of a linear model with 'soil type' as a fixed effect. The variation in native SOC decomposition rate (k_{SOC}) of unplanted soil and rhizosphere priming effect (RPE) across time and soil column depth respectively represent the parameters from the first and second series of incubations. RPE values were standardized to a common high predictor value across treatments. Root density values from the biogeochemical characterization of soil are used here to reflect *in situ* root density. See Supplementary Fig. 1 for abbreviations. ***, $P < 0.001$; **, $P < 0.01$; *, $P < 0.05$; †, $P < 0.10$. P values are derived from a two-sided Spearman's correlation test.

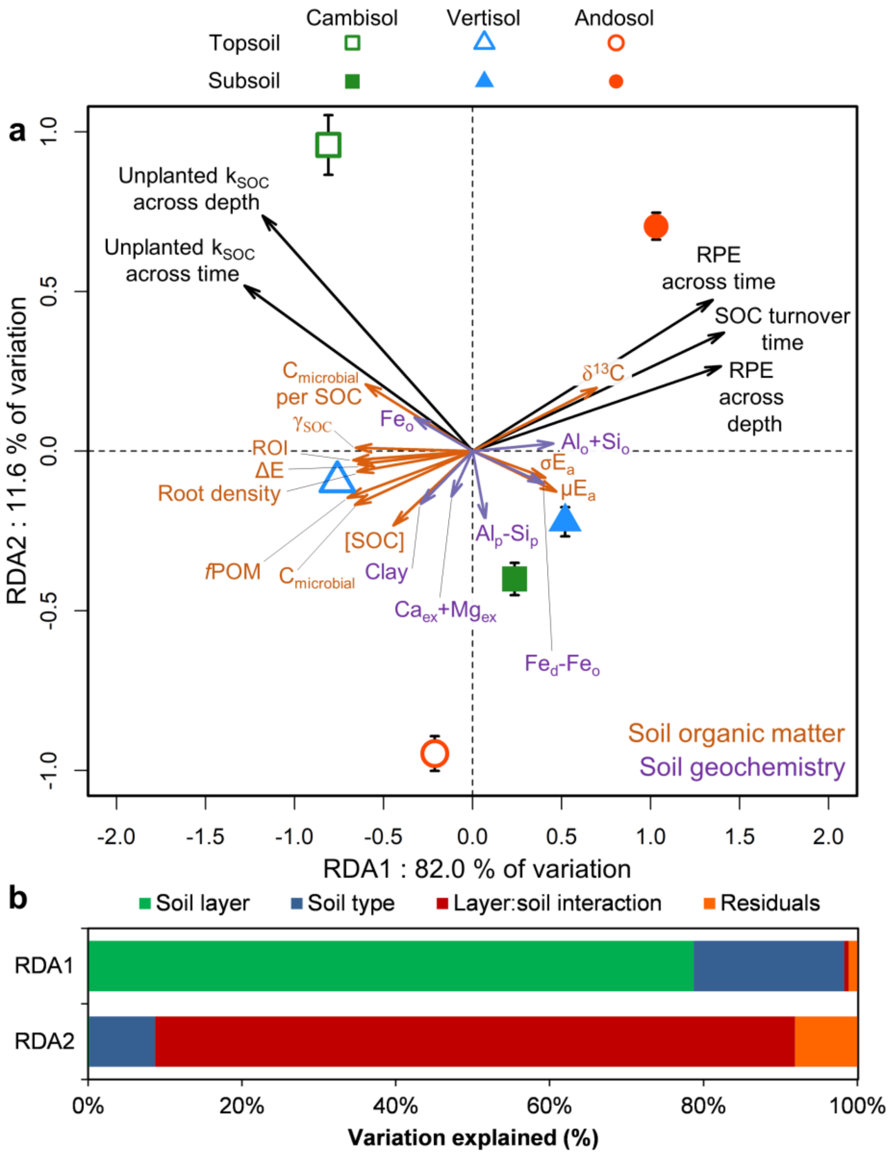

Supplementary Fig. 4. Soil biogeochemical drivers of soil organic carbon (SOC) dynamics. **a**, redundancy analysis of SOC dynamics variables (dark arrows) constrained by soil organic matter (SOM) properties (orange arrows) and soil geochemical properties (purple arrows, *n* = 24 soil cores/microcosms). We found that SOC dynamics properties was strongly related to soil biogeochemical properties ($F_{17,6}$ = 25.8, P < 0.001). The first axis of the redundancy analysis (RDA1) explained a large portion of variation (82.0 %, $F_{1,19}$ = 1154.6, P < 0.001), whereas the second axis explained only 11.6 % ($F_{1,19}$ = 163.7, P = 0.042). F and P values are derived from a two-sided ANOVA-like permutation test. The coordinate means of each treatment are plotted and error bars represent ± standard errors (*n* = 4 replicate soil cores). The variation in unplanted k_{SOC} and RPE across time and depth respectively represent the parameters from the first and second series of incubations. RPE values were standardized to a common high predictor value across treatments. Root density values from the biogeochemical characterization of soil are used here to reflect *in situ* root density. k_{SOC}, native SOC decomposition rate; RPE, rhizosphere priming effect; [SOC], soil organic carbon content; *f*POM, fraction of particulate organic matter; ΔE, energy density of SOC; μE_a and σE_a, mean and standard deviation of activation energy of decomposition; ROI, return-on-energy-investment; γ_{SOC}, degree of reduction of soil organic carbon; ex, d, o, and p, exchangeable, dithionite, oxalate, and pyrophosphate extracts, respectively. **b**, variance partitioning of axis values across experimental factors.

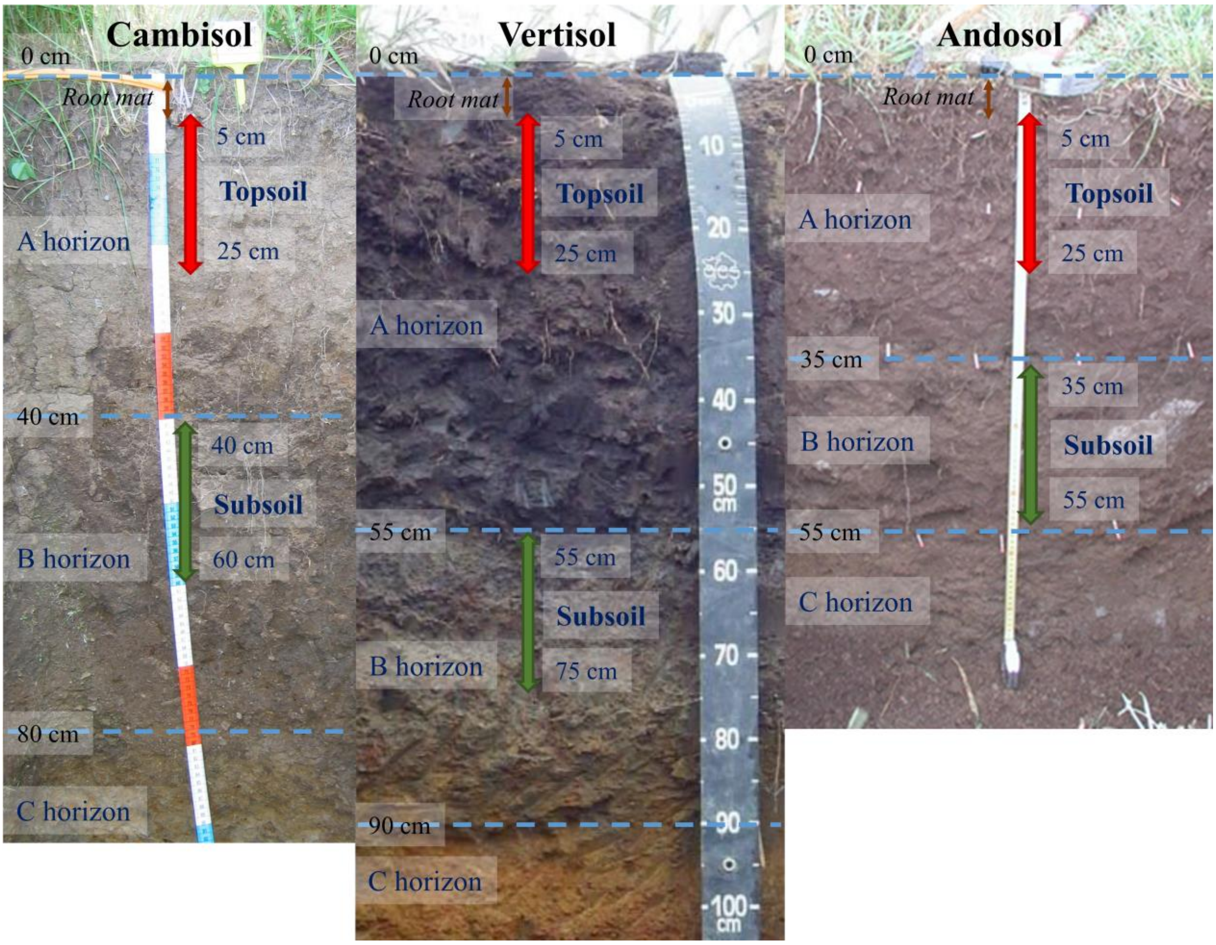

Supplementary Fig. 5. Soil profiles and core sampling design across the three soil types.

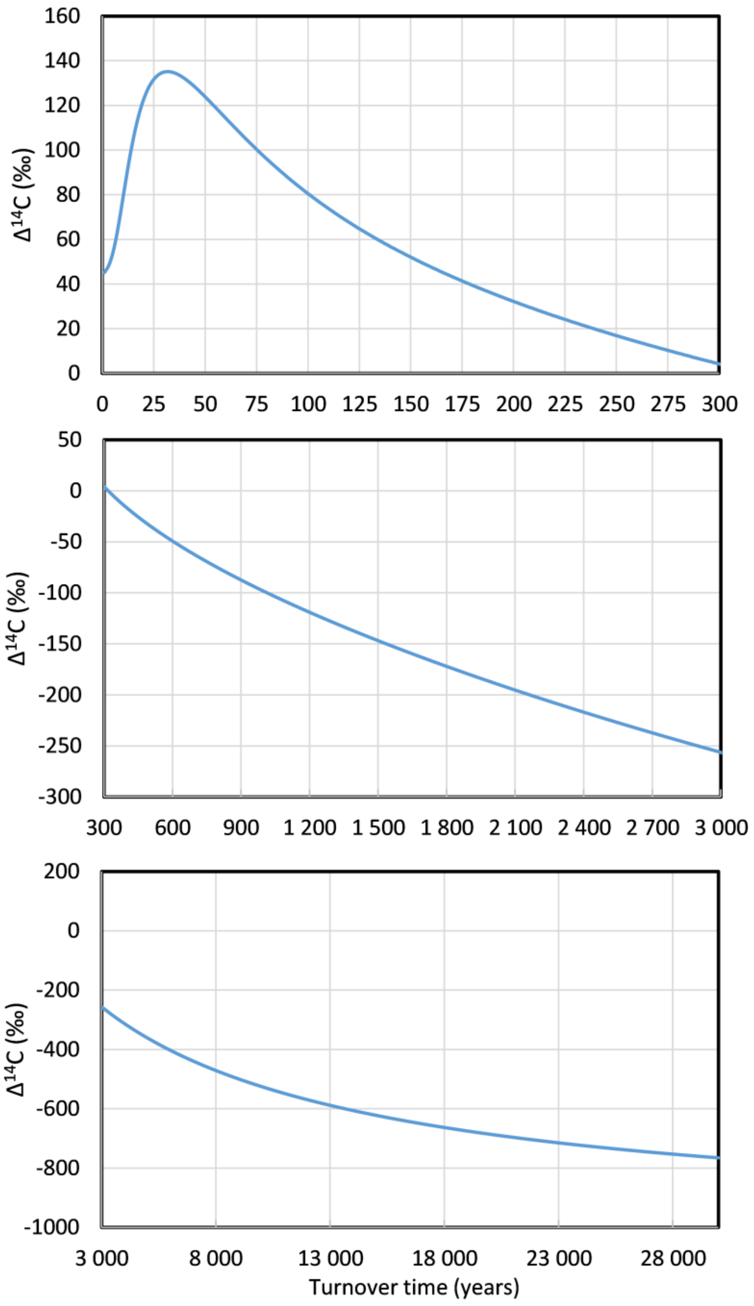

Supplementary Fig. 6. Relationship between turnover time and $\Delta^{14}C$ in year 2016 generated by a homogeneous one-pool model radiocarbon model assuming steady-state conditions.

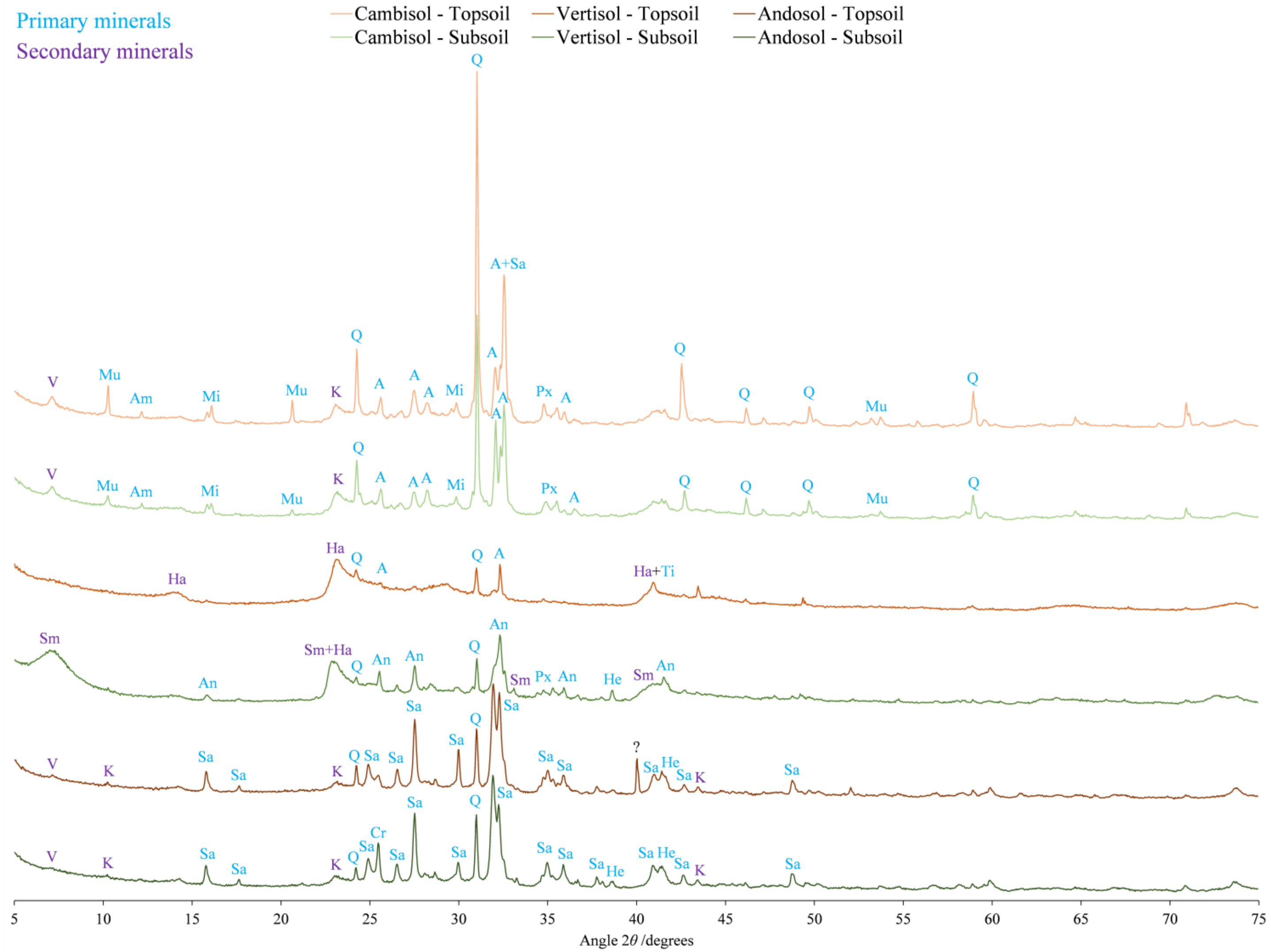

Supplementary Fig. 7. X-ray diffractograms (Co-Kα radiation) of samples from each soil horizon. All diffractograms are presented on the same vertical scale. Secondary minerals: Ha, Halloysite; K, kaolinite; Sm, smectite; V, vermiculite. Primary minerals: A, Albite; An, Andesine; Am, Amphibole; Cr, Cristobalite; He, Hematite; Mi, Microcline; Mu, Muscovite; Px, Pyroxene; Q, quartz; Sa, Sanidine; Ti, Titanomagnetite.

Supplementary Tables

Supplementary Table 1. Soil properties among soil types and layers. Mean ± standard error (*n* = 4 replicate soil cores).

	Cambisol		Vertisol		Andosol	
	Topsoil	Subsoil	Topsoil	Subsoil	Topsoil	Subsoil
Depth (cm)	5–25 cm	40–60 cm	5–25 cm	55–75 cm	5–25 cm	35–55 cm
**Soil organic matter**						
SOC concentration (g C kg^{-1} soil)	36.8 ± 1.6	26.1 ± 0.4	127.5 ± 7.4	19.8 ± 0.2	92.3 ± 4.6	58.2 ± 7.6
*f*POM (% SOC)	6.4 ± 0.5	1.9 ± 0.1	10.1 ± 0.4	2.6 ± 0.5	8.4 ± 1.4	3.5 ± 0.3
*f*MAOM (% SOC)	93.6 ± 0.5	98.1 ± 0.1	89.9 ± 0.4	97.4 ± 0.5	91.6 ± 1.4	96.5 ± 0.3
$\delta^{13}C$ (‰)	-26.6 ± 0.0	-25.5 ± 0.0	-27.4 ± 0.1	-27.1 ± 0.1	-26.5 ± 0.0	-25.0 ± 0.2
$\Delta^{14}C$ (‰)	+10.6 ± 7.2	-182.3 ± 6.9	-234.1 ± 13	-650.2 ± 0.9	-4.0 ± 11.0	-295.0 ± 36
SOC turnover time (years)	278 ± 29	1,933 ± 88	2,665 ± 192	16,987 ± 67	346 ± 46	3,780 ± 705
Energy density (kJ mol^{-1} SOM)	158.6 ± 3.1	111.9 ± 4.1	190.9 ± 5.7	127.7 ± 7.0	175.7 ± 3.0	140.6 ± 5.0
γ_{SOC}	2.86 ± 0.05	2.10 ± 0.07	3.23 ± 0.09	2.13 ± 0.12	3.08 ± 0.05	2.54 ± 0.07
HI (g $H_xC_y\cdot kg^{-1}$ SOC)	163.5 ± 6.1	109 ± 2.0	298.1 ± 8.0	75.3 ± 1.9	225.8 ± 5.1	159.3 ± 5.1
OI (g $O_2\cdot kg^{-1}$ SOC)	219.1 ± 4.5	288.9 ± 2.8	142.4 ± 1.4	140.1 ± 2.2	178.7 ± 0.9	237.6 ± 13
μE_a (kJ mol^{-1} SOM)	157.8 ± 0.1	158.4 ± 0.0	161.3 ± 0.3	165.9 ± 0.5	159.5 ± 0.2	161.5 ± 0.2
σE_a (kJ mol^{-1} SOM)	15.90 ± 0.00	15.93 ± 0.00	16.36 ± 0.08	18.04 ± 0.22	16.04 ± 0.03	16.41 ± 0.04
T_{90}-H_xC_y-pyrolysis (°C)	521.0 ± 1.7	532.8 ± 0.8	522.9 ± 0.8	532.3 ± 1.6	520.1 ± 1.0	533.3 ± 2.4
T_{50}-CO_2-pyrolysis (°C)	383.9 ± 0.7	386.3 ± 0.3	390.4 ± 0.8	406.3 ± 1.1	387.4 ± 1.0	390.5 ± 1.3
T_{50}-CO_2-oxidation (°C)	413.0 ± 0.4	419.3 ± 0.5	428.1 ± 2.1	460.0 ± 4.0	426.4 ± 0.2	434.3 ± 1.3
Return-on-Energy-Investment	1.01 ± 0.02	0.71 ± 0.03	1.18 ± 0.04	0.77 ± 0.04	1.10 ± 0.02	0.87 ± 0.03
$C_{microbial}$ (g C kg^{-1} soil)	0.67 ± 0.06	0.21 ± 0.01	1.99 ± 0.10	0.34 ± 0.03	1.08 ± 0.11	0.42 ± 0.01
$C_{microbial}$ per SOC (g C kg^{-1} SOC)	18.2 ± 0.9	8.2 ± 0.5	15.6 ± 0.7	17.1 ± 1.2	11.7 ± 0.7	7.4 ± 0.8
Root density (g dm^{-3})	1.54 ± 0.30	0.19 ± 0.07	6.71 ± 0.85	0.16 ± 0.05	1.37 ± 0.73	0.07 ± 0.01
**Soil geochemistry**						
Clay (%)	24.8 ± 0.8	32.6 ± 0.3	61.4 ± 4.0	60.5 ± 3.3	10.6 ± 1.0	3.8 ± 0.2
Phyllosilicate composition	K, V	K, V	H	H, S	K, V	K, V
Ca_{ex}+Mg_{ex} ($cmol^+$ kg^{-1} soil)	12.3 ± 0.4	17.1 ± 0.2	28.7 ± 0.5	41.1 ± 1.6	3.4 ± 0.7	1.5 ± 0.3
Fe_{d-o} (g kg^{-1} soil)	8.1 ± 0.2	13.1 ± 0.4	1.6 ± 0.2	3.6 ± 0.1	13.8 ± 0.4	13.2 ± 0.5
Fe_o (g kg^{-1} soil)	17.7 ± 0.3	21.5 ± 0.2	19.3 ± 0.9	6.9 ± 0.2	15.8 ± 0.4	17.1 ± 0.3
Al_o+Si_o (g kg^{-1} soil)	9.5 ± 0.4	10.6 ± 0.1	11.4 ± 1.1	4.0 ± 0.1	35.4 ± 0.7	46.5 ± 2.3
Al_p-xSi_p (g kg^{-1} soil)	1.2 ± 0.0	0.9 ± 0.0	-3.0 ± 1.4	-1.1 ± 0.1	10.7 ± 0.2	9.4 ± 0.2

SOC, soil organic carbon; *f*POM, fraction of particulate organic matter; *f*MAOM, fraction of mineral-associated organic matter; HI, hydrogen index; OI, oxygen index; γ_{SOC}, degree of reduction of SOC; μE_a and σE_a, mean and standard deviation of activation energy of decomposition; T_{90}-H_xC_y-pyrolysis, temperature at which 90 % of H_xC_y was evolved during pyrolysis; T_{50}-CO_2-pyrolysis and T_{50}-CO_2-oxidation, temperatures at which 50 % of CO_2 was evolved during pyrolysis and oxidation, respectively; $_{ex}$, $_{d}$, $_{o}$, and $_{p}$, exchangeable, dithionite, oxalate, and pyrophosphate extracts, respectively. Phyllosilicate composition: K, kaolinite; S, smectite; V, vermiculite. See Supplementary Figure 7 for XRD analyses of phyllosilicate composition.

Supplementary Table 2. Statistical results of analyses of variance for soil properties and incubations (*n* = 24 soil cores/microcosms).

	Soil layer			Soil type			Interaction		
	% of r^2	$F_{1,18}$	P	% of r^2	$F_{2,18}$	P	% of r^2	$F_{2,18}$	P
SOC content	45.5	171.8	**<0.001*** **	27.7	54.8	**<0.001*** **	51.5	56.8	**<0.001*** **
*f*POM *f*MAOM	85.5	110.6	**<0.001*** **	9.7	6.3	**0.008** **	4.8	3.1	0.068†
$\delta^{13}C$	35.2	88.9	**<0.001*** **	55.4	70.0	**<0.001*** **	9.4	11.8	**<0.001*** **
$\Delta^{14}C$	46.3	473.1	**<0.001*** **	49.5	253.0	**<0.001*** **	4.3	21.9	**<0.001*** **
SOC turnover time	31.2	684.8	**<0.001*** **	45.4	500.2	**<0.001*** **	23.4	257.1	**<0.001*** **
Energy density (ΔE)	79.0	147.3	**<0.001*** **	16.5	15.4	**<0.001*** **	4.5	4.2	**0.032***
γ_{SOC}	83.4	149.8	**<0.001*** **	9.7	8.7	**0.002** **	6.9	6.2	**0.008** **
HI	60.9	733.2	**<0.001*** **	11.8	71.2	**<0.001*** **	27.3	164.1	**<0.001*** **
OI	15.6	70.9	**<0.001*** **	75.5	171.2	**<0.001*** **	8.9	20.1	**<0.001*** **
μE_a	20.2	122.6	**<0.001*** **	70.0	212.0	**<0.001*** **	9.7	29.4	**<0.001*** **
σE_a	21.9	73.3	**<0.001*** **	55.0	92.3	**<0.001*** **	23.1	38.7	**<0.001*** **
T_{50}-CO_2-pyrolysis	23.8	91.3	**<0.001*** **	58.1	111.3	**<0.001*** **	18.0	34.5	**<0.001*** **
T_{50}-CO_2-oxidation	26.3	92.9	**<0.001*** **	58.3	102.8	**<0.001*** **	15.4	27.1	**<0.001*** **
T_{90}-H_xC_y-pyrolysis	97.8	88.2	**<0.001*** **	0.4	0.2	0.827^{ns}	14.4	0.8	0.460^{ns}
ROI	83.3	154.5	**<0.001*** **	11.8	11.0	**<0.001*** **	4.8	4.5	**<0.001*** **
$C_{microbial}$	58.1	303.8	**<0.001*** **	23.6	61.8	**<0.001*** **	18.2	47.6	**<0.001*** **
$C_{microbial}$:SOC	25.4	39.8	**<0.001*** **	43.6	34.2	**<0.001*** **	31.0	24.3	**<0.001*** **
Root density	43.3	62.7	**<0.001*** **	28.7	20.8	**<0.001*** **	28.0	20.3	**<0.001*** **
Clay	0.0	0.0	0.993^{ns}	98.2	302.2	**<0.001*** **	1.8	5.5	**0.013***
Ca_{ex}+Mg_{ex}	2.5	45.9	**<0.001*** **	93.1	863.5	**<0.001*** **	4.5	41.4	**<0.001*** **
Fe_{d-o}	4.8	62.5	**<0.001*** **	89.7	584.4	**<0.001*** **	5.5	36.1	**<0.001*** **
Fe_o	7.1	42.2	**<0.001*** **	33.4	99.2	**<0.001*** **	59.4	176.4	**<0.001*** **
Al_o+Si_o	0.3	3.2	0.091†	93.9	575.4	**<0.001*** **	5.9	35.9	**<0.001*** **
Al_p-xSi_p	0.0	0.1	0.820^{ns}	98.3	229.0	**<0.001*** **	1.7	3.9	**0.040***
$k_{SOC\ unplanted}$ - time‡	54.5	171.0	**<0.001*** **	31.2	48.9	**<0.001*** **	14.3	31.2	**<0.001*** **
RPE - time‡	63.9	752.9	**<0.001*** **	22.2	130.7	**<0.001*** **	13.9	81.8	**<0.001*** **
$k_{SOC\ unplanted}$ - depth¥	57.4	113.7	**<0.001*** **	24.3	24.3	**<0.001*** **	18.0	17.8	**<0.001*** **
RPE - depth¥	85.1	354.5	**<0.001*** **	7.6	15.8	**<0.001*** **	7.3	15.1	**<0.001*** **

SOC, soil organic carbon; *f*POM, fraction of particulate organic matter; *f*MAOM, fraction of mineral-associated organic matter; HI, hydrogen index; OI, oxygen index; γ_{SOC}, degree of reduction of SOC; μE_a and σE_a, mean and standard deviation of activation energy of decomposition; T_{90}-H_xC_y-pyrolysis, temperature at which 90 % of H_xC_y was evolved during pyrolysis; T_{50}-CO_2-pyrolysis and T_{50}-CO_2-oxidation, temperatures at which 50 % of CO_2 was evolved during pyrolysis and oxidation, respectively; $_{ex}$, $_{d}$, $_{o}$, and $_{p}$, exchangeable, dithionite, oxalate, and pyrophosphate extracts, respectively; $k_{SOC\ unplanted}$, native SOC decomposition rate of unplanted soil; RPE, rhizosphere priming effect. ‡, first series of incubations related to variation across time; ¥, second series of incubations related to variation across depth in the microcosms. F and P values are derived from a two-sided F-test.

Supplementary Table 3. Statistical results of analyses of covariance for the first series of incubations (variation across time, n = 288 and 144 incubations for k_{SOC} and RPE, respectively).

Factors	k_{SOC} ⁋			RPE		
	% of r^2	F	P	% of r^2	F	P
Plant-derived respiration (R_{plant})	51.5	578.3	**<0.001*** **	56.6	293.8	**<0.001*** **
Soil layer (Layer)	26.2	294.7	**<0.001*** **	0.3	1.5	0.216^{ns}
Soil type (Soil)	10.6	59.3	**<0.001*** **	0.1	0.2	0.845^{ns}
R_{plant}:Layer	10.3	116.2	**<0.001*** **	24.1	125.1	**<0.001*** **
R_{plant}:Soil	0.7	3.8	**0.025***	8.4	21.7	**<0.001*** **
Layer:Soil	0.0	0.3	0.779^{ns}	1.0	2.5	0.087†
R_{plant}:Layer:Soil	0.6	3.5	**0.034***	9.6	25.0	**<0.001*** **
Marginal r^2	0.84			0.80		
Conditional r^2	0.87			0.81		

⁋ log-transformed. k_{SOC}, native SOC decomposition rate; RPE, rhizosphere priming effect. F and P values are derived from a two-sided F-test.

Supplementary Table 4. Statistical results of analyses of covariance for the second series of incubations (variation across soil column depth, n = 144 and 72 incubations for k_{SOC} and RPE, respectively).

Factors	k_{SOC}⁋			RPE		
	% of r^2	F	P	% of r^2	F	P
Living root density (Root)	66.1	182.0	**<0.001*** **	60.8	142.8	**<0.001*** **
Soil layer (Layer)	10.3	28.4	**<0.001*** **	7.9	18.5	**<0.001*** **
Soil type (Soil)	10.5	14.4	**<0.001*** **	4.0	4.7	**0.014* **
Root:Layer	1.5	4.2	**0.044* **	15.6	36.7	**<0.001*** **
Root:Soil	6.8	9.3	**<0.001*** **	8.2	9.7	**<0.001*** **
Layer:Soil	3.0	4.5	**0.022* **	1.3	1.5	0.234^{ns}
Root:Layer:Soil	1.8	2.4	0.094†	2.1	2.5	0.096†
Marginal r^2		0.64			0.84	
Conditional r^2		0.88			0.87	
Root-derived respiration (R_{root})	43.1	141.2	**<0.001*** **	49.7	48.7	**<0.001*** **
Soil layer (Layer)	17.0	55.5	**<0.001*** **	5.2	5.1	**0.028* **
Soil type (Soil)	13.8	22.6	**<0.001*** **	8.1	4.0	**0.024* **
R_{root}:Layer	7.2	23.6	**<0.001*** **	14.4	14.1	**<0.001*** **
R_{root}:Soil	11.3	18.5	**<0.001*** **	17.8	8.7	**<0.001*** **
Layer:Soil	2.9	4.8	**0.014* **	2.6	1.3	0.287^{ns}
R_{root}:Layer:Soil	4.7	7.6	**<0.001*** **	2.3	1.1	0.337^{ns}
Marginal r^2		0.73			0.78	
Conditional r^2		0.85			0.78	
Net rhizodeposition (NetRhiz)	50.4	175.0	**<0.001*** **	51.4	34.1	**<0.001*** **
Soil layer (Layer)	10.4	36.0	**<0.001*** **	10.5	7.0	**0.011* **
Soil type (Soil)	7.2	12.5	**<0.001*** **	0.2	0.1	0.234^{ns}
NetRhiz:Layer	8.8	30.5	**<0.001*** **	24.6	16.3	**<0.001*** **
NetRhiz:Soil	17.1	29.6	**<0.001*** **	10.7	3.5	**0.036* **
Layer:Soil	5.7	9.8	**<0.001*** **	0.3	0.1	0.234^{ns}
NetRhiz:Layer:Soil	0.6	1.0	0.386^{ns}	2.3	0.8	0.234^{ns}
Marginal r^2		0.72			0.65	
Conditional r^2		0.74			0.65	

⁋ log-transformed for the predictors ’Living root density’ and ’Root-derived respiration’. k_{SOC}, native SOC decomposition rate; RPE, rhizosphere priming effect. F and P values are derived from a two-sided F-test.

Supplementary Table 5. Statistical results of two-way analyses of covariance for radiocarbon data in the second series of incubations (variation across soil column depth, *n* = 24 incubations).

Factors	$\Delta^{14}C_{soil}$			Mean age of respired SOC		
	% of r^2	F	P	% of r^2	F	P
Living root density (Root)	95.6	61.9	**<0.001*** **	93.7	72.1	**<0.001*** **
Soil type (Soil)	4.3	5.6	**0.034***	4.1	6.2	**0.027***
Root:Soil	0.1	0.0	0.972^{ns}	2.2	1.7	0.220^{ns}
Marginal r^2		0.85			0.81	
Conditional r^2		0.90			0.94	

$\Delta^{14}C_{soil}$ is the $\Delta^{14}C$ of respired soil organic carbon (SOC). F and P values are derived from a two-sided F-test.

Supplementary Table 6. Information about root C amount relative to soil organic carbon (SOC). Mean ± standard error (n = 4 replicate microcosms).

	Cambisol		Vertisol		Andosol	
	Topsoil	Subsoil	Topsoil	Subsoil	Topsoil	Subsoil
Pre-existing root C content (g C kg^{-1} soil)	0.84 ± 0.13	0.11 ± 0.04	6.01 ± 0.91	0.09 ± 0.03	1.20 ± 0.00	0.08 ± 0.02
Proportion of pre-existing root C in SOC at the beginning of the experiment (%)	2.26 ± 0.38	0.40 ± 0.15	4.43 ± 0.42	0.44 ± 0.15	1.19 ± 0.60	0.15 ± 0.04
Proportion of pre-existing root C lost by decomposition by the end of the experiment (%)	87.3 ± 3.3		91.0 ± 2.8		92.2 ± 4.9	
Proportion of pre-existing root C in SOC at the end of the experiment (%)	0.29 ± 0.08		0.42 ± 0.13		0.10 ± 0.06	

Supplementary Table 7. Site characteristics for each soil type.

	Cambisol	Vertisol	Andosol
Location	Theix	Saint Jean de Nay	Laqueuille
Latitude	45°43’24”N	45°04’43”N	45°38’20”N
Longitude	03°01’15”E	03°43’25”E	2°44’28”E
Elevation (m)	880	920	1040
Mean annual temperature (°C)	9.0	8.1	7.7
Mean annual precipitation (mm)	760	692	859
Parent material	granite	basalt	trachyandesite
Soil depth (m)	1	2.5	0.8
Soil texture	Loam	Clay	Silt

Supplementary Table 8. Additional soil properties. Mean ± standard error (n = 4 replicate soil cores).

	Cambisol		Vertisol		Andosol	
	Topsoil	Subsoil	Topsoil	Subsoil	Topsoil	Subsoil
SOC molar mass (g SOC mol^{-1} SOM)	6.11 ± 0.02	5.86 ± 0.01	6.51 ± 0.01	6.59 ± 0.01	6.28 ± 0.00	6.09 ± 0.06
Energy density (kJ g^{-1} SOC)	25.9 ± 0.4	19.1 ± 0.7	29.3 ± 0.8	19.4 ± 1.1	28.0 ± 0.5	23.1 ± 0.7
SOM C:N ratio (g C·g^{-1} N)	9.2 ± 0.1	9.3 ± 0.1	13.4 ± 0.3	13.9 ± 0.5	9.9 ± 0.1	10.7 ± 0.3
pH	5.8 ± 0.1	6.9 ± 0.0	5.9 ± 0.1	6.4 ± 0.0	5.3 ± 0.1	6.1 ± 0.1
CEC (cmol+ kg^{-1} soil)	13.4 ± 0.4	18.8 ± 0.3	31.4 ± 0.5	45.1 ± 1.7	6.0 ± 0.4	4.2 ± 0.2
Soil bulk density (kg dm^{-3})	0.81 ± 0.03	0.81 ± 0.03	0.51 ± 0.01	0.84 ± 0.03	0.54 ± 0.02	0.44 ± 0.06

SOC, soil organic carbon; SOM, soil organic matter; CEC, cation exchange capacity.

Supplementary Table 9. Information about soil N fertilization for planted treatments. Mean ± standard error (n = 4 replicate microcosms).

	Cambisol		Vertisol		Andosol	
	Topsoil	Subsoil	Topsoil	Subsoil	Topsoil	Subsoil
Initial soil mineral N content (mg kg^{-1})	20.9 ± 1.9	3.8 ± 0.3	31.0 ± 1.9	0.7 ± 0.2	49.4 ± 9.1	6.4 ± 0.6
Initial soil mineral N stock (g m^{-2})	13.0 ± 1.7	2.3 ± 0.3	11.9 ± 0.6	0.4 ± 0.2	19.9 ± 3.0	2.2 ± 0.5
Mineral N added (g m^{-2})		11.5		11.5		23.0
Final⁋ soil mineral N stock (g m^{-2})	13.0 ± 1.7	13.8 ± 0.3	11.9 ± 0.6	11.9 ± 0.2	19.9 ± 3.0	25.2 ± 0.5
Living plant N content (mg kg^{-1})	6.0 ± 0.4	5.4 ± 0.3	6.7 ± 0.3	5.0 ± 0.5	6.7 ± 0.3	7.4 ± 0.8

⁋ Post-fertilization.

Supplementary Table 10. Plant biomass and $\delta^{13}C$ at the end of the experiment and uncertainty in ^{13}C isotopic partitioning of root biomass. Mean ± standard error (n = 4 replicate microcosms).

	Cambisol		Vertisol		Andosol	
	Surface	Deep	Surface	Deep	Surface	Deep
Shoot $\delta^{13}C$ (‰)	-51.15 ± 0.07	-50.90 ± 0.19	-50.52 ± 0.41	-50.73 ± 0.12	-51.25 ± 0.21	-49.82 ± 0.32
Dead and living root $\delta^{13}C$ (‰)	-47.33 ± 0.96	-50.33 ± 0.08	-44.25 ± 1.19	-50.35 ± 0.39	-48.28 ± 0.54	-47.93 ± 0.14
Living root $\delta^{13}C$ (‰)	-50.58 ± 0.07		-50.15 ± 0.41		-49.37 ± 0.21	
f_{living} (%)	86.4 ± 3.8		74.2 ± 5.3		95.3 ± 3.0	
$\sigma_{f_{living}}$ (%)	0.6 ± 0.0		0.6 ± 0.0		0.6 ± 0.0	
$\Delta f_{living} \vert \Delta\delta^{13}C_{living}$ (%)	3.6 ± 0.2		3.3 ± 0.3		4.2 ± 0.2	
$\Delta f_{living} \vert \Delta\delta^{13}C_{dead}$ (%)	0.6 ± 0.2		1.1 ± 0.2		0.2 ± 0.1	
Dead and living root biomass (g m^{-2})	872 ± 34	1,376 ± 91	1,347 ± 194	681 ± 160	1,409 ± 135	808 ± 78
Living root biomass (g m^{-2})	753 ± 45	1,376 ± 91	981 ± 101	681 ± 160	1,338 ± 126	808 ± 78
Shoot biomass (g m^{-2})	1,893 ± 173	2,012 ± 38	2,731 ± 207	1,733 ± 168	2,147 ± 127	1,713 ± 86
Living plant biomass (g m^{-2})	2,645 ± 203	3,388 ± 97	3,712 ± 196	2,414 ± 316	3,485 ± 227	2,521 ± 158
Living plant $\delta^{13}C$ (‰)	-50.99 ± 0.07	-50.66 ± 0.14	-50.42 ± 0.40	-50.61 ± 0.13	-50.53 ± 0.20	-49.23 ± 0.27

f_{living} is the proportion of living roots in the living and dead root mixture; $\sigma_{f_{living}}$ is the standard error of f_{living} related to sampling and analytical errors; $\Delta f_{living}|\Delta\delta^{13}C_{living}$ and $\Delta f_{living}|\Delta\delta^{13}C_{dead}$ are the variations in f_{living} to a 1 ‰ variation in $\delta^{13}C_{living}$ and $\delta^{13}C_{dead}$, respectively.

Supplementary Table 11. Uncertainty in ^{13}C isotopic partitioning of CO_2 fluxes for the first series of incubations. Mean ± standard error (n = 4 replicate microcosms).

Soil type	Soil layer	Plant presence	Days after planting	Season	‰ $\delta^{13}C_{total}$	‰ $\delta^{13}C_{soil}$	‰ $\delta^{13}C_{plant}$	% f_{soil}	% σ_{fsoil}	% $\Delta f_{soil}\vert\Delta\delta^{13}C_{soil}$	% $\Delta f_{soil}\vert\Delta\delta^{13}C_{plant}$	% $\Delta RPE\vert\Delta\delta^{13}C_{soil}$	% $\Delta RPE\vert\Delta\delta^{13}C_{plant}$
		**Mean**			-37.6	-25.5	-53.8	57.3	1.1	2.0	1.5	0.2	19.3
		**Min**			-48.7	-26.6	-54.4	18.3	0.4	0.6	0.2	0.0	2.5
		**1st quartile**			-44.6	-26.0	-54.1	32.3	0.6	1.1	0.7	0.1	6.0
		**Mediane**			-37.6	-25.8	-54.0	56.5	0.7	2.0	1.5	0.1	11.4
		**3st quartile**			-30.3	-24.8	-53.8	81.9	1.5	2.9	2.4	0.1	24.2
		**Max**			-27.1	-24.4	-52.7	95.9	2.1	3.5	2.9	1.1	85.8
Cambisol	Topsoil	Planted	76	Fall	-40.5 ± 0.5	-24.6 ± 0.5	-54.4 ± 0.1	46.5 ± 1.7	1.0 ± 0.0	1.6 ± 0.1	1.8 ± 0.1	0.1 ± 0.0	2.6 ± 0.3
			139	Winter	-43.0 ± 1.4			38.4 ± 4.6	0.8 ± 0.1	1.3 ± 0.2	2.1 ± 0.2	0.1 ± 0.0	4.5 ± 1.2
			174	Spring	-45.1 ± 0.3			31.2 ± 0.7	0.7 ± 0.0	1.0 ± 0.0	2.3 ± 0.0	0.1 ± 0.0	7.5 ± 0.5
			201	Spring	-45.5 ± 0.3			30.0 ± 1.2	0.7 ± 0.0	1.0 ± 0.0	2.3 ± 0.0	0.1 ± 0.0	12.2 ± 1.4
			242	Summer	-46.4 ± 0.8			26.8 ± 2.7	0.7 ± 0.0	0.9 ± 0.1	2.5 ± 0.1	0.1 ± 0.0	16.1 ± 1.0
			272	Summer	-44.2 ± 0.5			34.3 ± 1.8	0.8 ± 0.0	1.1 ± 0.1	2.2 ± 0.1	0.1 ± 0.0	11.9 ± 0.9
		Unplanted	83	Fall	-32.4 ± 0.4		-53.8 ± 0.1	73.1 ± 1.3	1.4 ± 0.0	2.5 ± 0.0	0.9 ± 0.0		
			146	Winter	-33.2 ± 1.1			70.6 ± 3.6	1.4 ± 0.1	2.4 ± 0.1	1.0 ± 0.1		
			181	Spring	-30.2 ± 0.4			80.8 ± 1.4	1.6 ± 0.0	2.8 ± 0.0	0.7 ± 0.0		
			209	Spring	-30.2 ± 0.7			80.9 ± 2.4	1.6 ± 0.0	2.8 ± 0.1	0.7 ± 0.1		
			230	Spring	-29.1 ± 0.4			84.5 ± 1.5	1.6 ± 0.0	2.9 ± 0.1	0.5 ± 0.1		
			251	Spring	-29.0 ± 0.2			84.8 ± 0.7	1.7 ± 0.0	2.9 ± 0.0	0.5 ± 0.0		
	Subsoil	Planted	83	Fall	-43.5 ± 0.9	-24.4 ± 0.4	-53.8 ± 0.1	35.8 ± 3.1	0.7 ± 0.0	1.2 ± 0.1	2.2 ± 0.1	0.0 ± 0.0	4.3 ± 1.0
			146	Winter	-44.5 ± 3.0			32.6 ± 9.8	0.7 ± 0.1	1.1 ± 0.3	2.3 ± 0.3	0.1 ± 0.0	10.9 ± 3.7
			181	Spring	-45.7 ± 0.5			28.3 ± 1.6	0.6 ± 0.0	1.0 ± 0.1	2.4 ± 0.1	0.1 ± 0.0	21.5 ± 1.6
			209	Spring	-46.5 ± 0.6			25.8 ± 1.9	0.6 ± 0.0	0.9 ± 0.1	2.5 ± 0.1	0.1 ± 0.0	32.7 ± 2.1
			230	Spring	-48.7 ± 0.5			18.3 ± 1.5	0.6 ± 0.0	0.6 ± 0.1	2.8 ± 0.1	0.1 ± 0.0	52.4 ± 4.9
			251	Spring	-47.9 ± 0.5			20.7 ± 2.1	0.6 ± 0.0	0.7 ± 0.1	2.7 ± 0.1	0.1 ± 0.0	46.4 ± 2.7
		Unplanted	83	Fall	-35.3 ± 1.5		-53.8 ± 0.1	63.2 ± 5.0	1.1 ± 0.1	2.2 ± 0.2	1.3 ± 0.2		
			146	Winter	-31.6 ± 2.9			74.6 ± 8.8	1.2 ± 0.1	2.6 ± 0.3	0.9 ± 0.3		
			181	Spring	-31.3 ± 1.0			76.8 ± 3.4	1.2 ± 0.0	2.6 ± 0.1	0.8 ± 0.1		
			209	Spring	-33.4 ± 0.8			69.7 ± 2.6	1.1 ± 0.0	2.4 ± 0.1	1.0 ± 0.1		
			230	Spring	-27.4 ± 0.3			89.8 ± 1.1	1.4 ± 0.0	3.1 ± 0.0	0.3 ± 0.0		
			251	Spring	-29.1 ± 2.4			84.0 ± 8.1	1.3 ± 0.1	2.9 ± 0.3	0.5 ± 0.3		
Vertisol	Topsoil	Planted	83	Fall	-40.0 ± 0.6	-26.0 ± 0.3	-53.9 ± 0.4	49.8 ± 1.6	0.8 ± 0.0	1.8 ± 0.1	1.8 ± 0.1	0.1 ± 0.0	2.5 ± 0.2
			146	Winter	-41.5 ± 1.6			44.6 ± 5.4	0.7 ± 0.1	1.6 ± 0.2	2.0 ± 0.2	0.1 ± 0.0	4.3 ± 1.1
			181	Spring	-43.2 ± 0.3			38.2 ± 1.0	0.7 ± 0.0	1.4 ± 0.0	2.2 ± 0.0	0.1 ± 0.0	6.3 ± 0.3
			209	Spring	-43.6 ± 0.4			36.9 ± 0.8	0.7 ± 0.0	1.3 ± 0.0	2.3 ± 0.0	0.1 ± 0.0	8.4 ± 0.2
			230	Spring	-44.4 ± 0.7			34.1 ± 1.9	0.6 ± 0.0	1.2 ± 0.1	2.4 ± 0.1	0.1 ± 0.0	10.9 ± 0.4
			251	Spring	-43.4 ± 0.7			37.7 ± 2.6	0.7 ± 0.0	1.4 ± 0.1	2.2 ± 0.1	0.1 ± 0.0	8.6 ± 0.1
		Unplanted	83	Fall	-31.2 ± 0.7		-53.8 ± 0.1	81.4 ± 2.5	1.1 ± 0.0	2.9 ± 0.1	0.7 ± 0.1		
			146	Winter	-29.6 ± 2.2			84.7 ± 5.5	1.1 ± 0.1	3.1 ± 0.3	0.6 ± 0.3		
			181	Spring	-27.7 ± 0.2			93.1 ± 1.1	1.2 ± 0.0	3.4 ± 0.0	0.2 ± 0.0		
			209	Spring	-28.5 ± 0.2			91.2 ± 0.5	1.2 ± 0.0	3.3 ± 0.0	0.3 ± 0.0		
			230	Spring	-27.8 ± 0.2			93.7 ± 0.6	1.2 ± 0.0	3.4 ± 0.0	0.2 ± 0.0		
			251	Spring	-27.6 ± 0.6			94.3 ± 2.2	1.2 ± 0.0	3.4 ± 0.1	0.2 ± 0.1		
	Subsoil	Planted	83	Fall	-42.2 ± 1.3	-26.1 ± 0.4	-54.0 ± 0.1	42.2 ± 4.3	0.9 ± 0.1	1.5 ± 0.2	2.1 ± 0.2	0.0 ± 0.0	5.0 ± 1.0
			146	Winter	-47.4 ± 1.8			23.6 ± 6.3	0.7 ± 0.1	0.8 ± 0.2	2.7 ± 0.2	0.1 ± 0.0	18.3 ± 4.2
			181	Spring	-48.3 ± 0.2			20.4 ± 0.6	0.6 ± 0.0	0.7 ± 0.0	2.8 ± 0.0	0.1 ± 0.0	32.3 ± 1.2
			209	Spring	-48.3 ± 0.5			20.5 ± 1.6	0.6 ± 0.0	0.7 ± 0.1	2.8 ± 0.1	0.1 ± 0.0	38.8 ± 2.7
			230	Spring	-48.7 ± 0.7			19.0 ± 2.4	0.6 ± 0.0	0.7 ± 0.1	2.9 ± 0.1	0.1 ± 0.0	61.4 ± 7.1
			251	Spring	-46.5 ± 0.6			27.0 ± 2.0	0.7 ± 0.0	1.0 ± 0.1	2.6 ± 0.1	0.1 ± 0.0	36.2 ± 4.9
		Unplanted	83	Fall	-31.5 ± 0.8		-53.8 ± 0.1	80.2 ± 2.7	1.5 ± 0.0	2.9 ± 0.1	0.7 ± 0.1		
			146	Winter	-31.9 ± 1.5			79.0 ± 5.2	1.4 ± 0.1	2.8 ± 0.2	0.8 ± 0.2		
			181	Spring	-27.8 ± 0.7			93.8 ± 2.7	1.7 ± 0.0	3.4 ± 0.1	0.2 ± 0.1		
			209	Spring	-30.3 ± 1.3			84.8 ± 4.8	1.5 ± 0.1	3.1 ± 0.2	0.5 ± 0.2		
			230	Spring	-27.1 ± 0.8			95.9 ± 2.5	1.7 ± 0.0	3.5 ± 0.1	0.2 ± 0.1		
			251	Spring	-30.7 ± 3.8			81.2 ±	1.5 ± 0.2	3.0 ± 0.4	0.8 ± 0.4		
Andosol	Topsoil	Planted	83	Fall	-42.7 ± 0.5	-26.6 ± 0.5	-54.0 ± 0.2	41.0 ± 2.3	1.0 ± 0.0	1.5 ± 0.1	2.2 ± 0.1	0.0 ± 0.0	4.9 ± 0.9
			146	Winter	-43.4 ± 1.7			38.5 ± 6.1	1.0 ± 0.1	1.4 ± 0.2	2.2 ± 0.2	0.1 ± 0.0	7.0 ± 1.6
			181	Spring	-45.4 ± 0.3			31.3 ± 1.4	0.8 ± 0.0	1.1 ± 0.0	2.5 ± 0.0	0.1 ± 0.0	10.7 ± 1.0
			209	Spring	-45.4 ± 0.6			31.2 ± 2.0	0.8 ± 0.0	1.1 ± 0.1	2.5 ± 0.1	0.1 ± 0.0	18.1 ± 0.7
			230	Spring	-46.1 ± 0.7			28.7 ± 2.0	0.8 ± 0.0	1.1 ± 0.1	2.6 ± 0.1	0.1 ± 0.0	18.7 ± 1.6
			251	Spring	-45.9 ± 0.6			29.4 ± 2.4	0.8 ± 0.0	1.1 ± 0.1	2.6 ± 0.1	0.1 ± 0.0	17.4 ± 1.2
		Unplanted	83	Fall	-33.1 ± 0.1		-53.8 ± 0.1	76.2 ± 0.4	1.7 ± 0.0	2.8 ± 0.0	0.9 ± 0.0		
			146	Winter	-30.1 ± 2.6			83.2 ± 6.1	1.9 ± 0.1	3.2 ± 0.4	0.8 ± 0.4		
			181	Spring	-29.4 ± 0.2			89.5 ± 0.8	2.0 ± 0.0	3.3 ± 0.0	0.4 ± 0.0		
			209	Spring	-30.4 ± 0.2			86.1 ± 0.6	1.9 ± 0.0	3.2 ± 0.0	0.5 ± 0.0		
			230	Spring	-28.1 ± 0.1			94.5 ± 0.2	2.1 ± 0.0	3.5 ± 0.0	0.2 ± 0.0		
			251	Spring	-28.2 ± 0.5			94.1 ± 1.7	2.1 ± 0.0	3.5 ± 0.1	0.2 ± 0.1		
	Subsoil	Planted	83	Fall	-40.0 ± 0.6	-25.6 ± 0.5	-52.7 ± 0.3	46.9 ± 2.0	1.1 ± 0.0	1.7 ± 0.1	2.0 ± 0.1	0.2 ± 0.0	3.0 ± 0.3
			146	Winter	-40.5 ± 1.9			45.1 ± 6.8	1.1 ± 0.1	1.7 ± 0.3	2.0 ± 0.3	0.2 ± 0.0	4.2 ± 1.2
			181	Spring	-42.8 ± 1.3			36.5 ± 4.3	0.9 ± 0.1	1.4 ± 0.2	2.3 ± 0.2	0.2 ± 0.1	8.8 ± 1.9
			209	Spring	-43.8 ± 1.7			32.8 ± 6.2	0.9 ± 0.1	1.2 ± 0.2	2.5 ± 0.2	0.3 ± 0.1	16.5 ± 4.8
			230	Spring	-45.4 ± 0.8			26.9 ± 2.8	0.8 ± 0.0	1.0 ± 0.1	2.7 ± 0.1	0.7 ± 0.2	41.7 ± 7.8
			251	Spring	-46.4 ± 0.2			23.0 ± 1.5	0.7 ± 0.0	0.8 ± 0.0	2.9 ± 0.0	1.1 ± 0.2	85.8 ± 4.8
		Unplanted	83	Fall	-34.8 ± 0.8		-53.8 ± 0.1	67.4 ± 2.9	1.4 ± 0.1	2.4 ± 0.1	1.2 ± 0.1		
			146	Winter	-33.8 ± 1.8			71.1 ± 6.2	1.5 ± 0.1	2.5 ± 0.2	1.0 ± 0.2		
			181	Spring	-33.2 ± 0.7			72.9 ± 2.5	1.5 ± 0.0	2.6 ± 0.1	1.0 ± 0.1		
			209	Spring	-33.5 ± 1.4			71.9 ± 4.9	1.5 ± 0.1	2.5 ± 0.2	1.0 ± 0.2		
			230	Spring	-30.4 ± 0.5			82.9 ± 1.8	1.7 ± 0.0	2.9 ± 0.1	0.6 ± 0.1		
			251	Spring	-32.9 ± 2.2			74.3 ± 7.6	1.5 ± 0.1	2.6 ± 0.3	0.9 ± 0.3		

$\delta^{13}C_{total}$, $\delta^{13}C_{soil}$ and $\delta^{13}C_{plant}$ are the $\delta^{13}C$ of respectively total, native soil organic carbon (SOC) and plant-derived organic carbon respiration. f_{soil} is the proportion of CO_2 from native SOC respiration; σ_{fsoil} is the standard error of f_{soil} related to sampling and analytical errors; $\Delta f_{soil}|\Delta\delta^{13}C_{soil}$ and $\Delta f_{soil}|\Delta\delta^{13}C_{plant}$ are the variations in f_{soil} to a 1 ‰ variation in $\delta^{13}C_{soil}$ and $\delta^{13}C_{plant}$, respectively; $\Delta RPE|\Delta\delta^{13}C_{soil}$ and $\Delta RPE|\Delta\delta^{13}C_{plant}$ are the variations in RPE to a 1 ‰ variation in $\delta^{13}C_{soil}$ and $\delta^{13}C_{plant}$, respectively.

Supplementary Table 12. Uncertainty in ^{13}C isotopic partitioning of CO_2 fluxes for the second series of incubations. Mean ± standard error (n = 4 replicate soil cores).

Soil type	Soil layer	Depth	‰ $\delta^{13}C_{total}$	$\delta^{13}C_{soil}$	$\delta^{13}C_{plant}$	% f_{soil}	σ_{fsoil}	$\Delta f_{soil}\|\Delta\delta^{13}C_{soil}$	$\Delta RPE\|\Delta\delta^{13}C_{plant}$
		**Mean**	-37.1	-25.5	-50.4	53.3	1.2	1.9	12.2
		**Min**	-42.9	-26.6	-51.2	30.3	0.7	0.8	0.4
		**1st quartile**	-37.4	-25.8	-50.8	50.7	1.1	1.9	9.5
		**Mediane**	-37.4	-25.8	-50.8	50.7	1.1	1.9	9.5
		**3st quartile**	-36.6	-24.6	-50.0	57.4	1.4	2.2	14.1
		**Max**	-30.4	-24.4	-48.5	78.1	1.7	2.7	55.7
Cambisol	Topsoil	0-20 cm	-38.0 ± 0.4			49.7 ± 1.5	1.1 ± 0.0	1.9 ± 0.1	8.6 ± 0.5
		20-40 cm	-32.9 ± 1.6	-24.6 ± 0.5	-51.2 ± 0.1	68.9 ± 5.9	1.5 ± 0.1	1.2 ± 0.2	2.0 ± 0.1
		40-60 cm	-30.4 ± 1.3			78.1 ± 4.9	1.7 ± 0.1	0.8 ± 0.2	0.4 ± 0.4
	Subsoil	0-20 cm	-42.9 ± 0.2			30.3 ± 0.8	0.7 ± 0.0	2.6 ± 0.0	55.7 ± 3.6
		20-40 cm	-38.2 ± 0.8	-24.4 ± 0.4	-50.9 ± 0.1	48.2 ± 3.1	0.9 ± 0.0	2.0 ± 0.1	10.5 ± 0.8
		40-60 cm	-38.1 ±0.4			48.4 ± 1.4	0.9 ± 0.0	1.9 ± 0.0	9.8 ± 0.9
Vertisol	Topsoil	0-20 cm	-36.4 ± 0.0			57.9 ± 0.7	0.9 ± 0.0	1.7 ± 0.1	5.4 ± 0.5
		20-40 cm	-34.2 ± 1.9	-26.0 ± 0.3	-50.8 ± 0.4	66.8 ± 7.5	1.0 ± 0.1	1.3 ± 0.3	2.4 ± 0.5
		40-60 cm	-34.7 ± 2.1			64.8 ± 8.4	1.0 ± 0.1	1.4 ± 0.3	2.9 ± 0.9
	Subsoil	0-20 cm	-37.1 ± 0.1			55.5 ± 0.7	1.2 ± 0.0	1.8 ± 0.1	19.7 ± 2.5
		20-40 cm	-38.6 ± 1.6	-26.1 ± 0.4	-51.0 ± 0.4	49.4 ± 7.1	1.1 ± 0.1	2.0 ± 0.3	9.7 ± 2.0
		40-60 cm	-40.4 ± 2.1			42.3 ± 8.3	1.0 ± 0.1	2.3 ± 0.3	15.8 ± 6.1
Andosol	Topsoil	0-20 cm	-36.9 ± 0.0			55.8 ± 0.4	1.5 ± 0.0	1.9 ± 0.0	10.7 ± 1.4
		20-40 cm	-37.9 ± 0.7	-26.6 ± 0.5	-50.0 ± 0.2	51.7 ± 2.9	1.4 ± 0.1	2.1 ± 0.1	4.4 ± 0.5
		40-60 cm	-36.9 ± 1.4			55.9 ± 5.9	1.5 ± 0.1	1.9 ± 0.3	3.1 ± 0.3
	Subsoil	0-20 cm	-37.5 ± 0.6			48.1 ± 2.5	1.3 ± 0.1	2.3 ± 0.1	33.7 ± 4.7
		20-40 cm	-39.6 ± 0.9	-25.6 ± 0.5	-48.5 ± 0.1	39.0 ± 4.0	1.1 ± 0.1	2.7 ± 0.2	15.2 ± 0.5
		40-60 cm	-37.3 ± 1.5			48.8 ± 6.8	1.3 ± 0.1	2.2 ± 0.3	10.1 ± 0.6

$\delta^{13}C_{total}$, $\delta^{13}C_{soil}$ and $\delta^{13}C_{plant}$ are the $\delta^{13}C$ of respectively total, native soil organic carbon and root-derived organic carbon respiration. f_{soil} is the proportion of CO_2 from native SOC respiration; σ_{fsoil} is the standard error of f_{soil} related to sampling and analytical errors; $\Delta f_{soil}|\Delta\delta^{13}C_{plant}$ and $\Delta RPE|\Delta\delta^{13}C_{plant}$ are the variation in respectively f_{soil} and RPE to a 1 ‰ variation in $\delta^{13}C_{plant}$.

Supplementary Table 13. Uncertainty in ^{14}C isotopic partitioning for planted soil cores of the second series of incubations. Mean ± standard error (n = 4 replicate soil cores).

Soil type	Soil layer	Depth	mg C-CO_2 kg^{-1} soil day^{-1}	‰	mg C-CO_2 kg^{-1} soil day^{-1}	‰	mg C-CO_2 kg^{-1} soil day^{-1}	‰	‰	‰
			R_{total}	$\Delta^{14}C_{total}$	R_{plant}	$\Delta^{14}C_{plant}$	R_{soil}	$\Delta^{14}C_{soil}$	$\Delta\Delta^{14}C_{soil}\|\Delta\delta^{13}C_{plant}$	$\Delta\Delta^{14}C_{soil}\|\Delta\Delta^{14}C_{plant}$
Cambisol	Subsoil	0-20	41.1 ± 2.5	-761 ± 6	28.6 ± 1.8	-852 ± 3	12.5 ± 0.8	-551 ± 17	26.1 ± 1.0	4.6 ± 0.2
		40-60	9.7 ± 0.8	-622 ± 16	5.0 ± 0.5		4.7 ± 0.4	-377 ± 20	19.1 ± 0.6	2.1 ± 0.1
Andosol	Subsoil	0-20	45.3 ± 3.8	-731 ± 11	23.8 ± 3.3	-820 ± 7	21.5 ± 0.6	-633 ± 23	9.3 ± 2.1	2.2 ± 0.2
		40-60	14.3 ± 1.1	-654 ± 20	7.1 ± 0.4		7.2 ± 1.4	-452 ± 77	22.9 ± 11.4	2.4 ± 0.8
		Mean	27.6	-692	16.1	-836	11.5	-503	19.4	2.8

R_{total} and $\Delta^{14}C_{total}$ are respectively the total CO_2 flux and its $\Delta^{14}C$ of root-soil respiration. R_{plant} and $\Delta^{14}C_{plant}$ are respectively the the total CO_2 flux and $\Delta^{14}C$ of root-derived organic carbon respiration. R_{soil} and $\Delta^{14}C_{soil}$ are respectively the the total CO_2 flux and $\Delta^{14}C$ of soil organic carbon respiration. $\Delta\Delta^{14}C_{soil}|\Delta\delta^{13}C_{plant}$ and $\Delta\Delta^{14}C_{soil}|\Delta\Delta^{14}C_{plant}$ are the variations in $\Delta^{14}C_{soil}$ to a 1 ‰ variation in $\delta^{13}C_{plant}$ and to a 2 ‰ variation in $\Delta^{14}C_{plant}$, respectively.

Supplementary Table 14. Uncertainty in ^{13}C isotopic partitioning of soil organic carbon for the second series of incubations. Mean ± standard error (n = 4 replicate soil cores).

Soil type	Soil layer	Depth	‰			%		
			$\delta^{13}C_{total}$	$\delta^{13}C_{root}$	$\delta^{13}C_{soil}$	f_{root}	σ_{froot}	$\Delta f_{root}\|\Delta\delta^{13}C_{root}$
	**Mean**		-26.68	-50.41	-26.37	1.37	0.77	0.06
	**Min**		-28.22	-50.99	-27.36	0.00	0.51	0.00
	**1st quartile**		-27.4	-50.66	-26.96	0.37	0.52	0.02
	**Mediane**		-26.81	-50.57	-26.60	1.10	0.73	0.05
	**3st quartile**		-25.77	-50.42	-25.80	1.91	0.93	0.08
	**Max**		-25.06	-49.23	-25.03	4.98	1.17	0.22
Cambisol	Topsoil	0-20 cm	-27.08 ± 0.02	-50.99 ± 0.07	-26.66 ± 0.06	1.75 ± 0.09	0.56 ± 0.00	0.07 ± 0.00
		20-40 cm	-26.88 ± 0.04			0.93 ± 0.16	0.56 ± 0.00	0.04 ± 0.01
		40-60 cm	-26.73 ± 0.06			0.31 ± 0.27	0.56 ± 0.00	0.01 ± 0.01
	Subsoil	0-20 cm	-26.09 ± 0.07	-50.66 ± 0.14	-25.55 ± 0.02	2.17 ± 0.29	0.51 ± 0.01	0.09 ± 0.01
		20-40 cm	-25.66 ± 0.01			0.46 ± 0.06	0.51 ± 0.00	0.02 ± 0.00
		40-60 cm	-25.58 ± 0.03			0.11 ± 0.13	0.52 ± 0.00	0.00 ± 0.01
Vertisol	Topsoil	0-20 cm	-27.97 ± 0.06	-50.42 ± 0.40	-27.36 ± 0.14	2.67 ± 0.32	0.92 ± 0.01	0.12 ± 0.02
		20-40 cm	-27.66 ± 0.14			1.27 ± 0.62	0.93 ± 0.02	0.05 ± 0.03
		40-60 cm	-27.44 ± 0.21			0.35 ± 0.89	0.94 ± 0.02	0.02 ± 0.04
	Subsoil	0-20 cm	-28.22 ± 0.11	-50.61 ± 0.13	-27.06 ± 0.19	4.98 ± 0.51	0.90 ± 0.01	0.22 ± 0.02
		20-40 cm	-27.84 ± 0.07			3.34 ± 0.31	0.91 ± 0.01	0.14 ± 0.01
		40-60 cm	-27.40 ± 0.16			1.49 ± 0.70	0.92 ± 0.01	0.06 ± 0.03
Andosol	Topsoil	0-20 cm	-26.89 ± 0.01	-50.53 ± 0.20	-26.54 ± 0.01	1.54 ± 0.05	0.51 ± 0.00	0.07 ± 0.00
		20-40 cm	-26.72 ± 0.10			0.80 ± 0.45	0.51 ± 0.00	0.04 ± 0.02
		40-60 cm	-26.54 ± 0.01			0.00 ± 0.6	0.51 ± 0.00	0.00 ± 0.00
	Subsoil	0-20 cm	-25.38 ± 0.17	-49.23 ± 0.27	-25.03 ± 0.20	1.97 ± 0.60	1.16 ± 0.01	0.09 ± 0.03
		20-40 cm	-25.04 ± 0.03			0.08 ± 0.15	1.17 ± 0.01	0.09 ± 0.01
		40-60 cm	-25.12 ± 0.05			0.41 ± 0.20	1.17 ± 0.01	0.02 ± 0.01

$\delta^{13}C_{total}$, $\delta^{13}C_{soil}$ and $\delta^{13}C_{plant}$ are the $\delta^{13}C$ of respectively total, native and root-derived soil organic carbon. f_{root} is the proportion of root-derived soil organic carbon; σ_{froot} is the standard error of f_{root} related to sampling and analytical errors; $\Delta f_{root}|\Delta\delta^{13}C_{root}$ is the variation in f_{soil} to a 1 ‰ variation in $\delta^{13}C_{root}$.

Supplementary References

1. Wacker, L., Němec, M. & Bourquin, J. A revolutionary graphitisation system: Fully automated, compact and simple. *Nucl. Instrum. Methods Phys. Res. Sect. B Beam Interact. Mater. At.* **268**, 931–934 (2010).
2. Tisnérat-Laborde, N. *et al.* ECHoMICADAS: A new compact AMS system to measuring 14C for environment, climate and human sciences. in 16–20 (2015).
3. Synal, H.-A. Developments in accelerator mass spectrometry. *Int. J. Mass Spectrom.* **349–350**, 192–202 (2013).
4. Synal, H.-A., Stocker, M. & Suter, M. MICADAS: A new compact radiocarbon AMS system. *Nucl. Instrum. Methods Phys. Res. Sect. B Beam Interact. Mater. At.* **259**, 7–13 (2007).
5. Ruff, M. *et al.* On-line Radiocarbon Measurements of Small Samples Using Elemental Analyzer and MICADAS Gas Ion Source. *Radiocarbon* **52**, 1645–1656 (2010).
6. Tisnérat-Laborde, N., Poupeau, J. J., Tannau, J. F. & Paterne, M. Development of a Semi-Automated System for Routine Preparation of Carbonate Samples. *Radiocarbon* **43**, 299–304 (2001).
7. Trumbore, S. E., Sierra, C. A. & Hicks Pries, C. E. Radiocarbon Nomenclature, Theory, Models, and Interpretation: Measuring Age, Determining Cycling Rates, and Tracing Source Pools. in *Radiocarbon and Climate Change: Mechanisms, Applications and Laboratory Techniques* (eds. Schuur, E. A. G., Druffel, E. & Trumbore, S. E.) 45–82 (Springer International Publishing, 2016).
8. Stuiver, M. & Polach, H. A. Discussion Reporting of 14C Data. *Radiocarbon* **19**, 355–363 (1977).
9. Torn, M. S., Swanston, C. W., Castanha, C. & Trumbore, S. Storage and Turnover of Organic Matter in Soil. in *Biophysico-Chemical Processes Involving Natural Nonliving Organic Matter in Environmental Systems* (eds. Senesi, N., Xing, B. & Huang, P. M.) vol. 2 219–272 (Wiley & Sons Inc, 2009).
10. Sierra, C. A., Müller, M. & Trumbore, S. E. Modeling radiocarbon dynamics in soils: SoilR version 1.1. *Geosci Model Dev* **7**, 1919–1931 (2014).
11. Reimer, P. J. *et al.* The IntCal20 Northern Hemisphere radiocarbon age calibration curve (0–55 cal kBP). *Radiocarbon* 1–33 (2020) doi:10.1017/rdc.2020.41.
12. Hua, Q., Barbetti, M. & Rakowski, A. Z. Atmospheric Radiocarbon for the Period 1950–2010. *Radiocarbon* **55**, 2059–2072 (2013).
13. Disnar, J. R., Guillet, B., Keravis, D., Di-Giovanni, C. & Sebag, D. Soil organic matter (SOM) characterization by Rock-Eval pyrolysis: scope and limitations. *Org. Geochem.* **34**, 327–343 (2003).
14. Behar, F., Beaumont, V. & Penteado, H. L. D. B. Rock-Eval 6 Technology: Performances and Developments. *Oil Gas Sci. Technol. - Rev IFP* **56**, 111–134 (2001).
15. Khedim, N. *et al.* Topsoil organic matter build-up in glacier forelands around the world. *Glob. Change Biol.* **27**, (2020).
16. Hemingway, J. D., Rothman, D. H., Rosengard, S. Z. & Galy, V. V. Technical note: An inverse method to relate organic carbon reactivity to isotope composition from serial oxidation. *Biogeosciences* **14**, 5099–5114 (2017).
17. Hemingway, J. D. *rampedpyrox: Open-source tools for thermoanalytical data analysis*. (2017).
18. Bosatta, E. & Ågren, G. I. Soil organic matter quality interpreted thermodynamically. *Soil Biol. Biochem.* **31**, 1889–1891 (1999).
19. Leifeld, J. & von Lützow, M. Chemical and microbial activation energies of soil organic matter decomposition. *Biol. Fertil. Soils* **50**, 147–153 (2014).

20. Grant, K. E., Galy, V. V., Chadwick, O. A. & Derry, L. A. Thermal oxidation of carbon in organic matter rich volcanic soils: insights into SOC age differentiation and mineral stabilization. *Biogeochemistry* **144**, 291–304 (2019).
21. Hemingway, J. D. *et al.* Mineral protection regulates long-term global preservation of natural organic carbon. *Nature* **570**, 228–231 (2019).
22. Sanderman, J. & Grandy, A. S. Ramped thermal analysis for isolating biologically meaningful soil organic matter fractions with distinct residence times. *SOIL* **6**, 131–144 (2020).
23. Leifeld, J. Thermal stability of black carbon characterised by oxidative differential scanning calorimetry. *Org. Geochem.* **38**, 112–127 (2007).
24. Williams, E. K., Rosenheim, B. E., McNichol, A. P. & Masiello, C. A. Charring and non-additive chemical reactions during ramped pyrolysis: Applications to the characterization of sedimentary and soil organic material. *Org. Geochem.* **77**, 106–114 (2014).
25. Lafargue, E., Marquis, F. & Pillot, D. Rock-Eval 6 applications in hydrocarbon exploration, production, and soil contamination studies. *Rev. Inst. Fr. Pétrole* **53**, 421–437 (1998).
26. Espitalie, J., Deroo, G. & Marquis, F. La pyrolyse Rock-Eval et ses applications. Deuxième partie. *Rev. Inst. Fr. Pétrole* **40**, 755–784 (1985).
27. Carrie, J., Sanei, H. & Stern, G. Standardisation of Rock–Eval pyrolysis for the analysis of recent sediments and soils. *Org. Geochem.* **46**, 38–53 (2012).
28. Fernández, J. M., Peltre, C., Craine, J. M. & Plante, A. F. Improved Characterization of Soil Organic Matter by Thermal Analysis Using CO2/H2O Evolved Gas Analysis. *Environ. Sci. Technol.* **46**, 8921–8927 (2012).
29. Fernández, J. M., Plante, A. F., Leifeld, J. & Rasmussen, C. Methodological considerations for using thermal analysis in the characterization of soil organic matter. *J. Therm. Anal. Calorim.* **104**, 389–398 (2011).
30. Kucerik, J., Demyan, M. S. & Siewert, C. Practical application of thermogravimetry in soil science. *J. Therm. Anal. Calorim.* **123**, 2441–2450 (2016).
31. Werth, M. & Kuzyakov, Y. 13C fractionation at the root–microorganisms–soil interface: A review and outlook for partitioning studies. *Soil Biol. Biochem.* **42**, 1372–1384 (2010).
32. Cros, C., Alvarez, G., Keuper, F. & Fontaine, S. A new experimental platform connecting the rhizosphere priming effect with CO2 fluxes of plant-soil systems. *Soil Biol. Biochem.* **130**, 12–22 (2019).
33. Henneron, L., Cros, C., Picon-Cochard, C., Rahimian, V. & Fontaine, S. Plant economic strategies of grassland species control soil carbon dynamics through rhizodeposition. *J. Ecol.* **108**, 528–545 (2020).
34. Wang, R. *et al.* A novel 13C pulse-labelling method to quantify the contribution of rhizodeposits to soil respiration in a grassland exposed to drought and nitrogen addition. *New Phytol.* **230**, 857–866 (2021).
35. Phillips, D. L. & Gregg, J. W. Uncertainty in source partitioning using stable isotopes. *Oecologia* **127**, 171–179 (2001).
36. Schnyder, H. & Lattanzi, F. A. Partitioning Respiration of C3-C4 Mixed Communities Using the Natural Abundance 13C Approach - Testing Assumptions in a Controlled Environment. *Plant Biol.* **7**, 592–600 (2005).
37. Farquhar, G. D., Ehleringer, J. R. & Hubick, K. T. Carbon Isotope Discrimination and Photosynthesis. *Annu. Rev. Plant Physiol. Plant Mol. Biol.* **40**, 503–537 (1989).
38. Cheng, W. Measurement of rhizosphere respiration and organic matter decomposition using natural 13C. *Plant Soil* **183**, 263–268 (1996).
39. Cheng, W. & Dijkstra, F. A. Theoretical Proof and Empirical Confirmation of a Continuous Labeling Method Using Naturally 13C-Depleted Carbon Dioxide. *J. Integr. Plant Biol.* **49**, 401–407 (2007).

40. Pausch, J. & Kuzyakov, Y. Carbon input by roots into the soil: Quantification of rhizodeposition from root to ecosystem scale. *Glob. Change Biol.* **24**, 1–12 (2018).
41. Heath, J. *et al.* Rising atmospheric CO2 reduces sequestration of root-derived soil carbon. *Science* **309**, 1711–1713 (2005).
42. Phillips, R. P. *et al.* Roots and fungi accelerate carbon and nitrogen cycling in forests exposed to elevated CO2. *Ecol. Lett.* **15**, 1042–1049 (2012).
43. Dijkstra, F. A. & Cheng, W. Interactions between soil and tree roots accelerate long-term soil carbon decomposition. *Ecol. Lett.* **10**, 1046–1053 (2007).
44. Revelle, W. *psych: Procedures for Psychological, Psychometric, and Personality Research*. (2020).
45. Ben-Shachar, M. S., Lüdecke, D. & Makowski, D. Effectsize: estimation of effect size indices and standardized parameters. *J. Open Source Softw.* **5**, 2815 (2020).
46. Bates, D., Mächler, M., Bolker, B. & Walker, S. Fitting Linear Mixed-Effects Models Using lme4. *J. Stat. Softw. Vol 1 Issue 1 2015* (2015) doi:10.18637/jss.v067.i01.
47. Kuznetsova, A., Brockhoff, P. B. & Christensen, R. H. B. lmerTest Package: Tests in Linear Mixed Effects Models. *J. Stat. Softw.* **82**, 1–26 (2017).
48. Nakagawa, S. & Schielzeth, H. A general and simple method for obtaining R2 from generalized linear mixed-effects models. *Methods Ecol. Evol.* **4**, 133–142 (2013).
49. Barton, K. *MuMIn: Multi-model inference*. (2013).
50. Lefcheck, J. S. piecewiseSEM: Piecewise structural equation modeling in R for ecology, evolution, and systematics. *Methods Ecol. Evol.* **7**, 573–579 (2016).
51. Harrel, F. E. J. & Dupont, C. Hmisc: Harrell Miscellaneous. (2014).
52. Oksanen, J. *et al.* vegan: Community Ecology Package. (2013).
53. Legendre, P., Oksanen, J. & ter Braak, C. J. F. Testing the significance of canonical axes in redundancy analysis. *Methods Ecol. Evol.* **2**, 269–277 (2011).
54. R Core Team. *R: A Language and Environment for Statistical Computing*. (R Foundation for Statistical Computing, 2017).
