# Supplementary material for: Bioenergetic control of soil carbon dynamics across depth
Source: Nat Commun. 2022 Dec 12;13:7676. doi: 10.1038/s41467-022-34951-w (PMC9744916; doi:10.1038/s41467-022-34951-w)
Supplement: Supplementary file 3 — Reporting Summary [file 41467_2022_34951_MOESM3_ESM.pdf]

nature portfolio

Corresponding author(s): Ludovic Henneron

Last updated by author(s): Oct 31, 2022

Reporting Summary

Nature Portfolio wishes to improve the reproducibility of the work that we publish. This form provides structure for consistency and transparency in reporting. For further information on Nature Portfolio policies, see our Editorial Policies and the Editorial Policy Checklist.

Statistics

For all statistical analyses, confirm that the following items are present in the figure legend, table legend, main text, or Methods section.

n/a	Confirmed	
[ ]	[x]	The exact sample size (n) for each experimental group/condition, given as a discrete number and unit of measurement
[ ]	[x]	A statement on whether measurements were taken from distinct samples or whether the same sample was measured repeatedly
[ ]	[x]	The statistical test(s) used AND whether they are one- or two-sided<br>*Only common tests should be described solely by name; describe more complex techniques in the Methods section.*
[ ]	[x]	A description of all covariates tested
[ ]	[x]	A description of any assumptions or corrections, such as tests of normality and adjustment for multiple comparisons
[ ]	[x]	A full description of the statistical parameters including central tendency (e.g. means) or other basic estimates (e.g. regression coefficient) AND variation (e.g. standard deviation) or associated estimates of uncertainty (e.g. confidence intervals)
[ ]	[x]	For null hypothesis testing, the test statistic (e.g. F, t, r) with confidence intervals, effect sizes, degrees of freedom and P value noted<br>*Give P values as exact values whenever suitable.*
[x]	[ ]	For Bayesian analysis, information on the choice of priors and Markov chain Monte Carlo settings
[ ]	[x]	For hierarchical and complex designs, identification of the appropriate level for tests and full reporting of outcomes
[ ]	[x]	Estimates of effect sizes (e.g. Cohen's d, Pearson's r), indicating how they were calculated

Our web collection on statistics for biologists contains articles on many of the points above.

Software and code

Policy information about availability of computer code

Data collection	Radiocarbon analysis: BATS software; Rock-Eval analysis: Geoworks (Vinci Technologies, v1.4B4); TGA/DSC analysis: STARe (Mettler Toledo); X-ray diffraction: X'Pert Highscore Plus (Panalytical).
Data analysis	All statistical testing was carried out in the R statistical environment (Version 3.4.3) using the following packages: effectsize, Hmisc, lme4, lmerTest, MuMIn , piecewiseSEM, Psych and vegan. For the calculation of the activation energies of thermal decomposition, the thermograms of carbon evolved during pyrolysis and oxidation phases of Rock-Eval analysis were analysed with the software Python using the rampedpyrox package. For the determination of phyllosilicate mineralogy, X-ray diffractograms were analysed based on the PDF-2 database (ICDD) for phase identification.

For manuscripts utilizing custom algorithms or software that are central to the research but not yet described in published literature, software must be made available to editors and reviewers. We strongly encourage code deposition in a community repository (e.g. GitHub). See the Nature Portfolio guidelines for submitting code & software for further information.

Data

Policy information about availability of data

All manuscripts must include a data availability statement. This statement should provide the following information, where applicable:

- Accession codes, unique identifiers, or web links for publicly available datasets
- A description of any restrictions on data availability
- For clinical datasets or third party data, please ensure that the statement adheres to our policy

All data generated or analysed during this study are provided in the Supplementary Information (Supplementary Data). Additional data that support the findings of this study are available from the corresponding author (L.H.) upon request.

Human research participants

Policy information about studies involving human research participants and Sex and Gender in Research.

Reporting on sex and gender	*Use the terms sex (biological attribute) and gender (shaped by social and cultural circumstances) carefully in order to avoid confusing both terms. Indicate if findings apply to only one sex or gender; describe whether sex and gender were considered in study design whether sex and/or gender was determined based on self-reporting or assigned and methods used. Provide in the source data disaggregated sex and gender data where this information has been collected, and consent has been obtained for sharing of individual-level data; provide overall numbers in this Reporting Summary. Please state if this information has not been collected. Report sex- and gender-based analyses where performed, justify reasons for lack of sex- and gender-based analysis.*
Population characteristics	*Describe the covariate-relevant population characteristics of the human research participants (e.g. age, genotypic information, past and current diagnosis and treatment categories). If you filled out the behavioural & social sciences study design questions and have nothing to add here, write "See above."*
Recruitment	*Describe how participants were recruited. Outline any potential self-selection bias or other biases that may be present and how these are likely to impact results.*
Ethics oversight	*Identify the organization(s) that approved the study protocol.*

Note that full information on the approval of the study protocol must also be provided in the manuscript.

Field-specific reporting

Please select the one below that is the best fit for your research. If you are not sure, read the appropriate sections before making your selection.

☐ Life sciences ☐ Behavioural & social sciences ☒ Ecological, evolutionary & environmental sciences

For a reference copy of the document with all sections, see nature.com/documents/nr-reporting-summary-flat.pdf

Ecological, evolutionary & environmental sciences study design

All studies must disclose on these points even when the disclosure is negative.

Study description	This study combines a characterization of soil biogeochemical properties and a long-term soil incubation experiment in absence/presence of continuously 13C/14C-labelled plants. The experiment had a factorial design of two crossed factors: two soil layers including topsoil versus subsoil, and three soil types for a total of six treatments each including four replicates.
Research sample	The research sample consisted of 20 cm high soil cores collected from two layers of contrasting depth for each of three soil profiles studied. Soil cores collected for the incubation experiment were immediately proceeded in the field following sampling to establish microcosms with a new soil column made of intact soil cores derived exclusively from either topsoil or subsoil.
Sampling strategy	Intact columns of 8 cm diameter were extracted from the soil using a percussion core drill that can be opened from sideways to collect the soil cores. Four soil cores for soil biogeochemical characterization and twelve soil cores to estabish four microcosms for the soil incubation experiment were collected for each treatment.
Data collection	Ludovic Henneron, Sébastien Fontaine and Gaël Alvarez performed the soil sampling and processing. Ludovic Henneron and Sébastien Fontaine conducted most of soil biogeochemical characterization, except for radiocarbon analysis conducted by Christine Hatté, Rock-Eval analysis conducted by François Baudin, Pierre Barré and Lauric Cécillon, TGA-DSC analysis conducted by Alejandro Fernandez-Martinez, and pedogenic reactive metals analysis and X-ray diffraction conducted by Isabelle Basile-Doelsch. Ludovic Henneron and Sébastien Fontaine conducted all data acquisition of the soil incubation experiment, except for radiocarbon analysis conducted by Christine Hatté.
Timing and spatial scale	The soil cores were sampled between July and August 2016. Analyses for the soil biogeochemical characterization were performed

	within a few weeks or months after collection, expect for radiocarbon analyses performed in November 2017 and thermal analyses performed in November 2020. The soil incubation experiment was performed from August 2016 to July 2017 and the sampling of CO2 was perfomed throughout and at the end of the experiment. Analyses concerning the CO2 sampled were performed within a few weeks or months after collection, expect for radiocarbon analyses performed in November 2017.
Data exclusions	No data was excluded, with only a few exception for outliers shown in the figures.
Reproducibility	To ensure the lack of biais, all samples was proceeded in the same time within a single lab for each type of analysis.
Randomization	Samples were randomly processed for analyses
Blinding	Blinding was not relevant for this study.

Did the study involve field work? ☒ Yes ☐ No

Field work, collection and transport

Field conditions	For each site, sampling was perfomed under sunny conditions during summer time. No drought or flooding was occuring at the time of sampling. Climatic conditions of the sampling year can be found in Supplementary Table 7
Location	Precise information about the field site location can be found in Supplementary Table 7
Access & import/export	Site managers were contacted for their agreement concerning the soil sampling before accessing the sites. Sampled were identified and registered once back to the lab.
Disturbance	Disturbance due to soil sampling was modest at each site, and was minimized by recording of the sampling locations and refilling by additional soil.

Reporting for specific materials, systems and methods

We require information from authors about some types of materials, experimental systems and methods used in many studies. Here, indicate whether each material, system or method listed is relevant to your study. If you are not sure if a list item applies to your research, read the appropriate section before selecting a response.

Materials & experimental systems

n/a	Involved in the study	
☒	☐	Antibodies
☒	☐	Eukaryotic cell lines
☒	☐	Palaeontology and archaeology
☒	☐	Animals and other organisms
☒	☐	Clinical data
☒	☐	Dual use research of concern

Methods

n/a	Involved in the study	
☒	☐	ChIP-seq
☒	☐	Flow cytometry
☒	☐	MRI-based neuroimaging
